# Effect of *Bacillus velezensis* on *Aeromonas veronii*-Induced Intestinal Mucosal Barrier Function Damage and Inflammation in Crucian Carp (*Carassius auratus*)

**DOI:** 10.3389/fmicb.2019.02663

**Published:** 2019-11-15

**Authors:** Dong-Xing Zhang, Yuan-Huan Kang, Sheng Zhan, Ze-Lin Zhao, Sheng-Nan Jin, Chong Chen, Lei Zhang, Jin-Yu Shen, Chun-Feng Wang, Gui-Qin Wang, Xiao-Feng Shan, Ai-Dong Qian

**Affiliations:** ^1^College of Animal Science and Technology, Jilin Agricultural University, Changchun, China; ^2^Agriculture Ministry Key Laboratory of Healthy Freshwater Aquaculture, Key Laboratory of Fish Health and Nutrition of Zhejiang Province, Zhejiang Institute of Freshwater Fisheries, Huzhou, China

**Keywords:** *Bacillus velezensis*, Crucian carp, *Aeromonas veronii*, antibacterial activity, immune-gene expression, disease resistance

## Abstract

*Aeromonas veronii* is an emerging aquatic pathogen causing hemorrhagic septicemia in humans and animals. Probiotic is an effective strategy for controlling enteric infections through reducing intestinal colonization by pathogens. Here we report that the consumption of *Bacillus velezensis* regulated the intestinal innate immune response and decreased the degree of intestinal inflammation damage caused by the *A. veronii* in Crucian carp. In this study, we isolated four strains of *B. velezensis*, named C-11, S-22, L-17 and S-14 from apparently healthy Crucian carp, which exerted a broad-spectrum antimicrobial activity inhibiting both Gram-positive and Gram-negative bacteria especially the fish pathogens. *B. velezensis* isolates showed typical *Bacillus* characteristics by endospore staining, physiological and biochemical test, enzyme activity analysis (amylase, protease, and lipase), and molecular identification. Here, *Bacillus*-containing dietary was orally administrated to Crucian carp for 8 weeks before *A. veronii* challenge. Immunological parameters and the expression of immune-related genes were measured at 2, 4, 6, 8, and 10 weeks post-administration. The results showed that *B. velezensis* was found to promote the increase in the phagocytic activities of peripheral blood leukocytes (PBLs) and head kidney leukocytes (HKLs), as well as the increase in interleukin 1β (IL-1β), IL-10 and tumor necrosis factor α (TNF-α) concentration of serum. Lysozyme levels (113.76 U/mL), ACP activity (25.32 U/mL), AKP activity (130.08 U/mL), and SOD activity (240.63 U/mL) were maximum (*P* < 0.05) in the *B. velezensis* C-11 treated group at 8 week. Our results showed that Crucian carp fed with the diet containing *B. velezensis* C-11 and S-22 developed a strong immune response with significantly higher (*P* < 0.05) levels of IgM in samples of serum, mucus of skin and intestine compared to *B. velezensis* L-17 and S-14 groups. Moreover, *B. velezensis* spores appeared to show no toxicity and damage in fish, which could inhabit the gut of Crucian carp. *B. velezensis* restrained up-regulation of pro-inflammation cytokines (IL-1β, IFN-γ, and TNF-α) mRNA levels in the intestine and head kidney at final stage of administration, and the expression of IL-10 was increased throughout the 10-week trial. *A. veronii* infection increased the population of inflammatory cells in the intestinal villi in the controls. In contrast, numerous goblet cells and few inflammatory cells infiltrated the mucosa in the *B. velezensis* groups after challenge with *A. veronii*. Compared with *A. veronii* group, *B. velezensis* could safeguard the integrity of intestinal villi. The highest post-challenge survival rate (75.0%) was recorded in *B. velezensis* C-11 group. The present data suggest that probiotic *B. velezensis* act as a potential gut-targeted therapy regimens to protecting fish from pathogenic bacteria infection.

**IMPORTANCE:**

In this work, four *Bacillus velezensis* strains isolated from apparently healthy Crucian carp, which exhibited a broad-spectrum antibacterial activity especially the fish pathogens. Administration of *B. velezensis* induced the enhancement of the intestinal innate immune response through reducing intestinal colonization by pathogens. The isolation and characterization would help better understand probiotic can be recognized as an alternative of antimicrobial drugs protecting human and animal health.

## Introduction

Crucian carp (*Carassius auratus*) is the freshwater fish species with the largest production cultured in various areas of China (Rhee et al., 2014; Yang et al., 2018). However, Crucian carp aquaculture in China has been threatened by increasing outbreaks of bacterial diseases, causing tremendous economic losses. The motile *Aeromonas* species, especially *Aeromonas veronii* is recognized as a widespread fish pathogen that causes hemorrhagic septicemia, ulcerations of skin and gastrointestinal tract infections (Abu-Elala et al., 2015; Jagoda et al., 2017; Zepeda-Velázquez et al., 2017). However, widespread exposure of antibiotics remained in the aquaculture, which result in the emergence of drug-resistant pathogens and food safety concerns (Cabello et al., 2013; Dawood et al., 2016; Suez et al., 2018). There is increasing evidence that probiotics have emerged as preventive treatment for the invasion and colonization of pathogens (Akhter et al., 2015; Meidong et al., 2017b). Various microorganisms exert beneficial effects through controlling the balance of microbes in gut, enhancing growth performance and improving the ecosystem of gut microbiota have been considered as probiotics in aquaculture including *Lactobacillus, Bacillus* (Han et al., 2015; Kong et al., 2017), *Enterococcus* and *Vibrio* (Chen et al., 2015). Confirmation that probiotic administration could be an effective alternative to chemical therapies or vaccination was elucidated in fish affected by *Bacillus* (Hong et al., 2005; Khochamit et al., 2015). The genus *Bacillus* that form endospores have the capacity for surviving extreme conditions, such as low pHs and high temperatures.

Previous studies have demonstrated that *Bacillus* species such as *Bacillus subtilis* and *Bacillus velezensis* have been used as probiotics to control bacterial diseases in aquaculture since the species have capacity to produce versatile anti-microbial compounds as well as immune response promotion substance (Guo et al., 2016; Yi et al., 2018; Zaineldin et al., 2018). Research in African catfish (*Clarias gariepinus*) model confirmed that *Bacillus* species are appropriate candidates for aquaculture as delivery vehicles improving growth performance, serum antioxidants, and immune parameters (Reda et al., 2018). Thus, some *Bacillus* strains could be developed as potential probiotic and biological control agents. *Bacillus velezensis* was first proposed by Cristina et al. (2005) as a heterotypic synonym of *B. amyloliquefaciens*. Previous studies have demonstrated that *B. velezensis* exhibit considerable antagonistic against pathogen by producing versatile anti-microbial compounds, such as bacillomycin, surfactins, fengicins, and amylocyclicin (Palazzini et al., 2016; Fan et al., 2017). *B. velezensis* that possess antagonistic activity to *A. salmonicida* and *A. hydrophila* was isolated from marine recirculation aquaculture systems (RASs), and researchers have identified the structures of antimicrobial compounds from *B. velezensis*, which demonstrated that the isolate is an potential probiotic agent in *Oncorhynchus mykiss* (rainbow trout) (Gao et al., 2017). Genome analysis confirmed that secondary metabolite genes involved in the biosynthesis of anti-microbial compounds and D-galacturonate metabolism are enriched in *B. velezensis* (Hee et al., 2018). Of note, the limitations of antibiotics, vaccines, and probiotics in fish models suggest that aquaculture disease management should emphasize on harmless, preventative, and eco-friendly methods (Daniell et al., 2010).

The gastrointestinal tract serves as an important physiological barrier that uptake and processing of various antigens occurs primarily at the mucosal surface. The intestinal mucosal surface comprises several types of intestinal epithelial cells (IECs), which are mainly exposed to pathogenic or opportunistic bacteria, virus, and commensal (Groschwitz and Hogan, 2009; Caballero and Pamer, 2015). The dynamic interactions between mucosal barrier and these microorganisms can induce the inflammatory response and alterations in the function of the intestinal barrier (Honda and Dan, 2012). The mucosal surface of intestine is considered to be portals of entry used by pathogens in fish (Ringø et al., 2007). *A. veronii* can enter the fish through injury to the skin or gill, increasing colonization of opportunistic bacteria, and resulting in damage to the mucus cells (Parker and Shaw, 2011). Farming conditions alerted immune system in the gut, which increased transcript levels for immune-related genes of mid-intestine in Atlantic salmon, *Salmo salar* (Løkka et al., 2014). The intestinal homeostasis is disrupted, which exacerbated intestinal inflammation, leading to the decrease lysozyme activity and down-regulate expression of tight junction proteins of intestine following *A. hydrophila* infection in Jian carp (*Cyprinus carpio*) (Wu et al., 2017). Probiotic formulae containing spore-forming bacteria, which are proposed to prevent pathogen colonization by mechanisms that except for a described intestinal mucosal barrier function in aquatic animals remain poorly defined. Recently, the application of probiotics has concentrated in innate immunity, microbial community structure, and stress response in aquaculture (Zhou et al., 2016; Eissa et al., 2018). Probiotics mixture improved the non-specific immune parameters to maintain survival rate in a fish model infected with *A. sobria* (Reda et al., 2018). *B. licheniformis* inhibit pro-inflammatory cytokines TNF-α and IL-1β mRNA expression induced by *A. hydrophila* (Giri et al., 2017). Here, we speculated that probiotic could enhance the immune responses and intestinal epithelial barrier function. To further analyze this hypothesis, four potential probiotic *B. velezensis* were isolated from Crucian carp gut. The effects of *B. velezensis* strains on cytokines gene expression, intestinal mucosal barrier function, and disease resistance in Crucian carp were investigated. Therefore, the present study contributes to provide evidence that pathogen exclusion and protective effect in fish mediated by probiotic that enhances intestinal mucosal barrier function and inhibits pathogen colonization.

## Materials and Methods

### Experimental Fish

Crucian carp (length: 15.20 ± 1.3 cm, body weight: 55.20 ± 1.2 g) were supplied by HeiShui Aquaculture, Jilin, China. Fish were acclimatized in flow-through aquariums (200 L) at 26 ± 1.0°C, with natural photoperiod. Fish were fed the basal diet twice a day in the amount of 2% of their body weight for 2 weeks. During the whole experiment periods, the physicochemical parameters of the water were measured daily (5.6 ± 0.45 mg/L of dissolved oxygen, 0.12 ± 0.01 mg/L of ammonia, 0.015 ± 0.003 mg/L of nitrate and 7.8 ± 0.5 of pH). This study was conducted following the Jilin Agriculture University Institutional Animal Care and Use Committee (JLAU08201409), and the experimental procedures were performed in compliance with the National Institutes of Health guide for the care and use of Laboratory animals (NIH Publications No. 8023).

### Bacterial Isolation and Identification

Crucian carp were anesthetized with diluted tricaine methanesulfonate (MS-222, Sigma, United States) at the concentration of 100 mg/L. The whole intestines was dissected and homogenized in 10- fold dilution of sterile saline (0.85%) to remove intestinal contents. The supernatant was heated at 80°C for 15 min after centrifuged for 5 min at 400 × *g*, then serially diluted and spread on Luria-Bertani (LB) agar plates and incubated at 30°C for 24 h. According to the morphological features of *Bacillus* species, some colonies were re-streaked on LB agar plates twice and the purified strains were maintained in 80% glycerol broth (w/v) at −80°C. Genomic DNA was extracted from *Bacillus* isolates using EasyPure Genomic DNA Kit (Tiangen Biotech, China). The 16S rRNA gene segment was amplified using universal primers (Table 1), and all isolates were further confirmed by sequence analysis of housekeeping genes (DNA gyrase subunit B, *gyr*B and RNA polymerase beta subunit, *rpo*B) (Table 1). The amplified fragments were sequenced (TaKaRa, Dalian, China), and the sequences were used for alignment with Clustal-W and phylogenetic analysis by the neighbor-joining method of MEGA 7.0 software. The physiological and biochemical indicators for identification of *Bacillus* isolates were performed using API 50CH system (BioMérieux, Lyon, France), and the results were analyzed using the APILAB Plus software version 3.3.3.

**TABLE 1:** Sequence of oligonucleotide primers used for this study.

**Genes**	**Primers (5′ to 3′)**	**Accession no.**	**Use**
16S rRNA	F: AGAGTTTGATCMTGGCTCAG	FJ358616	Specific identification
	R: TACGGMTACCTTGTTACGACTT		
*gyr*B	F: ATCATACAGGAACGACGACAC	HQ844067	Specific identification
	R: CATTCTTGCTCTTGCCGCCA		
*rpo*B	F: AGCGTCGTCTGTCAGCATTAGG	CP010052	Specific identification
	R: CATAGAAGGACCGTCAGCAAGG		
IL-10	F: AACTGATGACCCGAATGGAAAC	HQ259106.1	Real-time-PCR
	R: CACCTTCTCCCAGTCGTCAAA		
IL-1β	F: AACTGATGACCCGAATGGAAC	AJ249137	Real-time-PCR
	R: CACCTTCTCCCAGTCGTCAAA		
IFN-γ	F: AACAGTCGGGTGTCGCAAG	EU909368	Real-time-PCR
	R: TCAGCAAACATACTCCCCAG		
TNF-α	F: TTATGTCGGTGCGGCCTTC	EU069817	Real-time-PCR
	R: AGGTCTTTCCGTTGTCGCTTT		
β-Actin	F: GATGCGGAAACTGGAAAGGG	AB039726	Real-time-PCR
	R: ATGAGGGCAGAGTGGTAGACG		

### Antimicrobial Assay

Agar well-diffusion protocol was performed based on Schillinger and Lucke method (Schillinger and Lücke, 1989) to determine the antimicrobial activity of all isolates against the pathogenic bacteria such as *Aeromonas veronii*, *Aeromonas hydrophila*, *Aeromonas caviae*, *Aeromonas salraonicida*, *Streptococcus agalactiae*, *Staphylococcus aureus*, *Escherichia coli, Salmonella Typhimurium*, *Salmonella choleraesuis*, *Clostridium perfringens*, *Proteus hauseri* were cultured in LB broth at 30 or 37°C for 18 h. The cultured bacteria was diluted to 10^8^ CFU/mL by LB broth, which were used to prepared agar plates with wells (3 mm in diameter), and then 3 μL cultures of *Bacillus* strains (10^8^ CFU/mL in LB broth) were placed in each of the empty well. After 18 h of incubation at 30°C, the diameter of the inhibition zones were measured in millimeters. The determination was repeated twice with duplicate samples.

### Analysis of Enzyme Activities

The cellulase activity was measured as previously reported (Dashtban et al., 2010). Briefly, the cultures of *Bacillus* strains were centrifuged at 7,000 × *g* for 10 min, and then 500 μL 1% (w/v) carboxymethyl cellulose (CMC) as substrate was added in 1 mL phosphate buffer saline (PBS, 0.1M, pH 6.8) with 500 μL supernatant and incubated at 37°C for 1 h. A boiled enzyme extract served as a blank control and we used D-glucose (0.01–0.06 μg/mL) as a standard. Reaction was stopped by adding 1.0 mL 0.03% (w/v) dinitrosalicylic acid (DNS), and followed by boiling the samples for 10 min. The absorbance of reaction mixture was recorded at 574 nm. One cellulase activity unit was defined as number of molecules of glucose released from cellulose mg^–1^ protein min^–1^ at 37°C.

The quantitative activity of amylase was measured using the 3, 5-dinitrosalicylic acid (DNS) method modified by Rick and Stegbauer (1974). One amylase activity unit was defined as the number of molecules of maltose released from starch mg^–1^ protein min^–1^ at 37°C. The reaction mixture recorded the absorbance at 540 nm.

Protease activity was measured by the modified method of Puri et al. (2002) using casein as a substrate and tyrosine as a standard. The sample (50 μL) was added to 150 μL 50 mM glycine NaOH buffer (pH 9.0) containing 2% (w/v) casein and incubated for 10 min at 50°C. Reaction was terminated by adding 200 μL 0.4M trichloroacetic acid (TCA) and the mixture was centrifuged at 8000 *g* for 10 min. The supernatant 50 μL was added to 250 μL 0.4M sodium carbonate (Na_2_CO_3_) and 50 μL Folin-Phenol reagent, and incubated for 30 min at 40°C. The absorbance at 680 nm was measured. One unit of protease activity is defined as the amount of enzyme required to liberate 1 μmole of tyrosine under defined standard assay conditions. All experiments were repeated three times.

### Growth Curves

Growth curves were measured using microplate reader (Molecular Devices, SpectraMax M2e) according to the method of Grangeon et al. (2017). Briefly, single colony of *Bacillus* strains were inoculated in LB broth and incubated at 30°C for 12 h. The bacterial cultures were adjusted to an optical density at 600 nm (OD_600_) of 0.2 in the LB medium grown at 30°C for 6 h, and then 100 μL of each suspension diluted to an OD_600_ of 0.05 was transferred to a 96-well plate. The bacteria were grown at 30°C for 12 h, and readings were determined hourly. Each experiment was conducted in triplicate.

### Resistances in Stimulated Conditions

Tolerance in stimulated conditions was estimated following the methods described by Ahire et al. (2011). The *Bacillus* cultures were grown at 30°C overnight in LB broth. The vegetative cells were inactivated by lysozyme (100 mg/mL) for 10 min and pelleted at 4,000 × *g* for 5 min. The spores were resuspended in PBS (10^8^ CFU/mL) after washed twice with sterile PBS. For high temperature resistance, the spores were heated at 80, 90, and 100°C by water bath for 5 and 10 min respectively. The overnight cultures of *Bacillus* strains were incubated in PBS of pH 1.0, 2.0, and 3.0 at 37°C for 2 h. Similarly, bile salt resistance of *Bacillus* strains were determined by inoculating the bacteria in simulated intestinal fluid (1 mg/mL of pancreatin (porcine pancreas; Sigma) and 2.5, 5.0, 7.5, and 10% bile salts) adjusted to pH 8.0 followed by incubation at 37°C for 2 h. Aliquots were drawn at different intervals for dilution plate count on LB agar plates. Percentage survival at each time interval was calculated by comparing to the viable cell number at 0 h. All experiments were repeated three times.

### Antibiotic Susceptibility Assay

The antibiotic susceptibility of *Bacillus* isolates was performed according to the method recommended by National Committee for Clinical Laboratory Standards (NCCLS) using antibiotic disks (Oxoid, England) (Jones, 1984), such as Ampicillin, Piperacillin, Cephalexin, Cefazolin, Cefradine, Cefuroxim, Ceftazidime, Amikacin, Gentamicin, Amikacin, Gentamicin, Kanamycin, Neomycin, Tetracycline, Doxycycline, Minocycline, Erythromycin, Norfloxacin, Ofloxacin, Ciprofloxacin, Polymyxin, Furazolidone, Chloramphenicol, Amoxicillin, and Clindamycin. In brief, overnight cultured bacterial suspensions were adjusted to 2 × 10^8^ CFU/mL and spread on Muller-Hinton agar (AppliChem, China). Antibiotic disks were dispensed on to the plates and incubated at 30°C for 24 h. Diameter of zones of inhibition were measured (mm), and the antibiotic sensitivity was recorded as different grades based on their activity. *Escherichia coli* ATCC 25922 and *S. aureus* ATCC 25923 were used as the control bacterial strains. Assays were performed in triplicate.

### Pathogenicity Test

The acute toxicity of *Bacillus* strains was determined as described by Guo et al. (2016). Briefly, the cultures of *Bacillus* isolates were grown at 30°C overnight in LB broth. The vegetative cells were treated with lysozyme (100 mg/mL) for 10 min and centrifuged at 4,000 × *g* for 5 min. The spores of bacterial pellets were resuspended in PBS (10^8^ CFU/mL) after washed twice with sterile PBS. Healthy Crucian carp were injected intraperitoneally with 100 μL of the identified strains and the control group was injected with the same volume of PBS. The abnormal behaviors and clinical symptoms of injected fish were monitored daily for 2 weeks.

For the chronic test, spores of the *Bacillus* isolates or PBS were sprayed on basal diet feed, which was oven-dried at 40°C for 6 h and containing a concentration of 10^8^ CFU/g feed to prepare probiotic supplemented and control diets (Supplementary Figure S1). Healthy Crucian carp were administrated with *Bacillus*-supplemented feed or control feed (2% of body weight daily) for 5 weeks. The mortality, abnormal behaviors and clinical symptoms of injected fish were monitored daily. The spleen, head kidney, liver, intestine, and heart were fixed in 4% neutralformalin and embedded in paraffin for histopathological analysis. Serial sections (5 μm thick) were stained with hematoxylin and eosin (H&E), for light microscopy analysis.

### Experimental Plan

The methods of probiotics added into feed were widely applied in the aquatic animal (Ghosh et al., 2016). In short, four *Bacillus* strains were grown in LB broth at 30°C for 24 h. Bacterial pellets (1 × 10^8^ CFU/g) washed by sterile PBS were thoroughly mixed with basal diet and then oven-dried at 40°C for 6 h (Supplementary Figure S1). Basal diet stirring mixed with sterile PBS was considered as control. The dried diet (approximately 1 g) of each *Bacillus* strain was homogenized in 9 mL sterile PBS, and centrifuged for 5 min at 400 × *g*. Then, the serial dilutions of supernatant of the fish intestine were prepared at a range of 10^–3^ to 10^–6^ using sterile PBS. Further, 100 μL from each dilution was spread on Luria-Bertani (LB) agar plates and incubated at 30°C for 24 h (Utiswannakul et al., 2011). The number of bacteria in each dilution was counted, and the suspected colonies were identified by endospore stain, biochemical tests and molecular identification. The prepared feed was stored at 4°C until use.

Six hundred healthy crucian carp juveniles (body weight: 55.20 ± 1.2 g) procured from a local aquaculture farm were acclimatized to laboratory conditions for 2 weeks in flow-through aquariums (200 L) at 26 ± 1.0°C, as described above. The fish were randomly divided into six experimental groups, with three replicates in each group (i.e., per group: 30 × 3 = 90 fish). The control group was fed on basal diet without probiotic supplementation, and the treatment groups were fed with *B. velezensis* coated basal diet (*B. velezensis* C-11, S-22, L-17, and S-14) at 3% of body weight twice daily (08:00 and 18:00 h) for 8 weeks. All groups fish were fed with normal basal diet till end of the trial. During the administration, the water and unconsumed feed were removed frequently with oxygen saturation, and temperature of water ranged from 26 to 31°C.

### Sample Collection

Fish were randomly collected from each group at 0, 2, 4, 6, 8, and 10 weeks post-administration. Blood samples were collected from the caudal vein using a 1 mL syringe after anesthetized with MS-222. Serum was obtained by centrifugation (2,000 × *g*, 10 min, 4°C) and stored at −80°C. The gut of Crucian carp was excised and lavaged with protease inhibitor buffer [PBS containing 1 × protease inhibitor cocktail, 1 mM phenylmethylsulfonyl fluoride (PMSF, Solarbio), 0.1 mg/mL soybean trypsin inhibitor (Solarbio), and 0.5% BSA (Solarbio); pH 7.2] (Zhang et al., 2010). The mucosal fluid was scraped from the surface of the intestine and transferred to an Eppendorf tube. After vigorous vortexing, the sample was centrifuged at 40 × *g* for 10 min at 4°C. Skin mucus was collected by gently scraping the skin surface and transferred into an Eppendorf tube. The mucus samples were vigorously vortexed, and centrifuged at 400 × *g* for 10 min at 4°C. Fish were carefully dissected, and then the pieces of head kidney (HK) and intestine were rapidly excised, frozen in liquid nitrogen, and stored at −80°C, until RNA extraction.

### Phagocytic Activity Assay

Peripheral blood leukocytes (PBLs) were isolated as previously described (Hani et al., 2004). Briefly, blood extracted from the caudal vein was diluted 10 fold with Leibovitz medium (L-15, Solarbio, China) supplemented with 100 U/mL penicillin, 100 ug/mL streptomycin, 25 U/mL heparin and 5% fetal calf serum (FCS). Blood cell suspensions were layered onto 51/34% discontinuous Percoll (Solarbio, China) cushions. Head kidney was excised and washed with RPMI-1640 medium (Solarbio, China), and transferred to RPMI-1640 medium supplemented with 100 U/mL penicillin, 100 ug/mL streptomycin and 5% FCS. Cell suspensions generated using a 100 μm steel mesh. Head kidney leukocytes (HKLs) suspensions were placed onto 30/51% discontinuous density gradients. All suspensions were centrifuged at 500 × *g* for 30 min at 4°C. The cells were harvested at the interface and washed twice with L-15 containing 5% FCS. Phagocytic activity of leukocytes were assessed as previously described (Geng et al., 2012). The above-prepared leukocytes (100 μL of 1 × 10^6^ cell/mL) from each fish in the different treatment groups were loaded onto sterile slide and allowed to adhere for 30 min at 25°C. Bacterial suspension of *S. aureus* (100 μL of 1 × 10^8^ cell/mL) was transferred to the cell monolayer and incubated at 25°C for 45 min. The unattached cells removed by washing three times with PBS. Within 15 min of slides being air-drying, which were fixed in methanol for 30 s and stained with Giemsa (Solarbio, China) for 10 min. Phagocytic activity is expressed as the percentage of cells calculated by enumerating 200 phagocytes under a microscope.

### Non-specific Immune Parameters

The serum samples collected at 2, 4, 6, 8, and 10 weeks were used for determining the lysozyme (LZY), acid phosphatase (ACP), alkaline phosphatase (AKP), superoxide dismutase (SOD), interleukin-1β (IL-1β), interleukin-10 (IL-10), and tumor necrosis factor-α (TNF-α) activity. The measurement was performed using ELISA kits (Nanjing Jiancheng Bioengineering Institute, China) according to the manufacturer’s instructions. Immunoglobulin M (IgM) in the serum, intestinal mucus and skin mucus was evaluated using ELISA Kit (Cusabio, China) as described in the manufacture’s protocol. All analyses were conducted in triplicates.

### Quantification of Spores in the Fish Intestine

To detect the survival of the *Bacillus* strains in the fish intestine, the gastrointestinal tract (approximately 4.5 g) of each group was homogenized in 4.5 mL sterile PBS as previously described (Galagarza et al., 2018). Then, the homogenization was heated at 80°C for 15 min after centrifuged for 5 min at 400 × *g*, and serial dilutions of supernatant of the fish intestine were prepared at a range of 10^–1^ to 10^–6^ using sterile PBS. Subsequently, 100 μL from each dilution was spread on Luria-Bertani (LB) agar plates and incubated at 30°C for 24 h (Utiswannakul et al., 2011). The number of bacteria in each dilution was counted, and the suspected colonies were identified with gram stain and endospore stain, and biochemical tests (i.e., propanediol, positive catalase, positive nitrite reduction, and variable oxidase).

Total DNA from suspected colonies was extracted using the EasyPure Genomic DNA Kit (Tiangen Biotech, China) according to the manufacture’s instructions. Then the real-time quantitative polymerase chain reaction (qRT-PCR) for specific 16S rRNA gene and DNA sequencing was performed. Specific primers to each of the *Bacillus* strains were used (Table 2). The PCR conditions were as follow: 95°C for 5 min, 40 cycles of denaturation at 95°C for 1 min, annealing at 60°C for 1 min, and extension at 72°C for 1 min using real-time PCR 7500 system (Applied Biosystem, United States).

**TABLE 2:** Primers specific for strains C-11, S-22, L-17, and S-14 of *Bacillus velezensis* for RT-qPCR analysis.

**Strain of *B. velezensis***	**Primers (5′ to 3′)**	**Amplicon size (bp)**	**Accession no.**
C-11	F: ACAAGTGCCGTTCA AATAGG	279	MN044920.1
	R: TTCGCCACTGGT GTTCC		
S-22	F: AGGCAGCAGTAGGGA ATCTT	160	MN044921.1
	R: GCCGTGGCTTTCTGGTTA		
L-17	F: GGGGAGGGTCATTGGAA	194	MN044922.1
	R: CGTTTACGGCGTGG ACTA		
S-14	F: GCTAACGCATTAAGC ACTCC	231	MN044923.1
	R: AACCCAACATCTCAC GACAC		

### RNA Isolation and Real-Time Quantitative PCR

Total RNA was extracted from the head kidney and intestine using High Pure RNA Tissue Kit (Tiangen Biotech, China) following the manufacturer’s instructions. The quantity of all RNA samples were examined using Nanodrop 2000c (Thermo Scientific, United States), and cDNA was synthesized by Reverse Transcriptase M-MLV Kit (Takara, Japan) according to the manufacturer’s instructions. The synthesized cDNA was used as a template for qRT-PCR analysis. qRT-PCR was performed using 7500 system (Applied Biosystem, United States) to quantify the relative expression levels of IL-1β, IL-10, TNF-α and IFN-γ, and β-actin was used as the internal control gene quantified by qRT-PCR. Each reaction was conducted in a final volume of 20 μL containing 10 μL SYBR Green Master Mix (Takara, Japan), 8 μL of sterile water, 0.5 μL of both forward and reverse primers (Table 1), and 1 μL of cDNA. Each sample was determined in triplicate. Data were analyzed using the 2^–Δ^ ^Δ^ ^CT^ method was performed as described by Livak and Schmittgen (2001).

### Challenge Test

Virulent *A. veronii* TH0426 (GenBank: CP012504.1) was grown in LB broth at 30°C for 18 h, harvested by centrifugation (2000 × *g*, 4°C for 20 min), washed and resuspended in PBS. Two days after the last bleeding, fish were anesthetized by bath immersion with 60 mg/L MS-222 for 10 min before challenge. The fish from the probiotics treated groups and the control group were orally intubated with 200 μL of viable *A. veronii* (1 × 10^7^ CFU/mL) per fish. Fish orally intubated with 200 μL PBS were used as the blank control group. In this study, the dose and concentration of *A. veronii* had been verified based on a preliminary experiment. The daily mortality of the challenged fish was monitored for 2 weeks. The relative percentage survival (RPS,%) was calculated as follow: RPS = (1 -% mortality in treated group/% mortality in control group) × 100.

### Histological Assessment

After challenge, the mid-intestinal tracts were fixed in 4% neutral formalin and embedded in paraffin for histopathological analysis. Serial sections (5 μm thick) were stained with hematoxylin and eosin (H&E), for light microscopy analysis. The intestinal parameter from six fish was expressed as the mean villus length (μm) and width (μm) for each group. The goblet cells (number per villus) of 10 selected villi per section were measured. Moreover, the number of inflammatory cells was calculated from six microscope vision of each fish and the data were converted to an area of 1 mm^2^.

### Statistical Analysis

All statistical analyses were performed using SPSS v.16.0 software and GraphPad PRISMM v7.0. One-way analysis of variance (ANOVA) followed by Tukey’s test were used to analyze differences between the treatments. Differences between experimental groups were considered as significant at *P* < 0.05. Data are presented as the mean ± standard deviation (SD).

## Results

### Isolation and Identification of Probiotic *Bacillus* spp.

**FIGURE 1:**
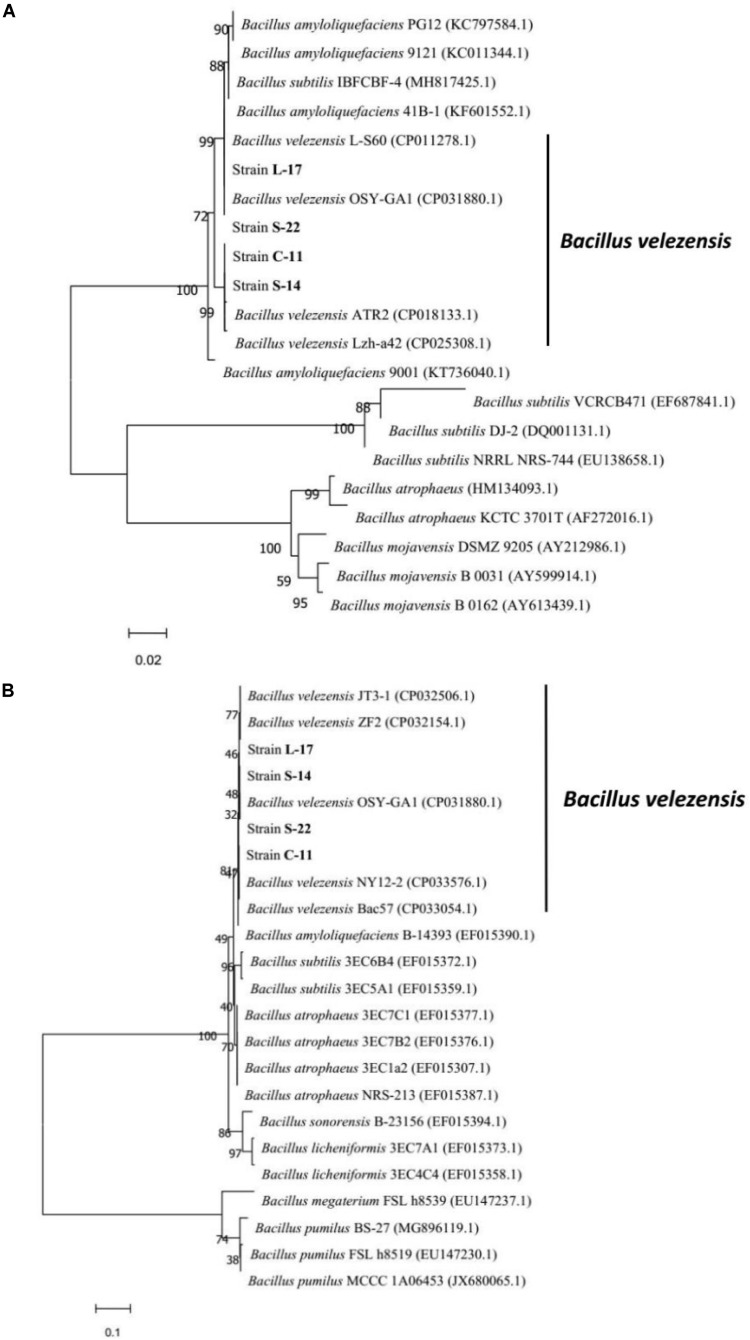
Phylogenetic tree of the closely related species in *Bacillus* genus based on *gyr*B **(A)** and *rpo*B **(B)** sequences. The sequences were aligned using Clustal W tool in MEGA 7.0, and phylogenetic analysis was performed using the neighbor-joining method. The bootstrap percentages, based on 10,000 replications, are shown. The scale bars indicate 0.02 or 0.1 substitutions per nucleotide position. The gene accession numbers are shown in the parentheses after each species.

A total of 136 spore-forming bacteria were isolated from 113 fish gastrointestinal tract samples based on heat treatment. These strains appeared gram-positive, rod-shaped, motile, and endospore-forming (Supplementary Figure S2). White and wet colonies with 3–4 mm diameter were observed on LB agar plates (Supplementary Figure S2A). The results suggested that the isolates were capable of producing catalase, amylase and utilizing glucose, which were similar to *Bacillus* spp. (Supplementary Table S1). Among the isolates, 60 out of 136 isolates exhibited antagonistic activity against the aquatic pathogens (*A. veronii* and *A. hydrophila*). The bacteriocin-like activity of *Bacillus* isolates were determined by agar well-diffusion method and the results showed that four isolates display inhibitory activities against fish pathogenic bacteria, including *A. caviae*, *A. salraonicida*, *E. coli*, *S. Typhimurium*, *S. choleraesuis*, and *P. hauseri*, especially *A. veronii, A. hydrophila* (Supplementary Table S2). Antagonistic effects were also observed on 3 g-positive pathogens: *S. aureus*, *S. agalactiae*, and *C. perfringens*. As shown in Supplementary Figure S3, strain C-11, S-22, L-17, and S-14 have stronger ability of pathogen inhibition. Partial 16S rRNA sequences of the isolates demonstrated that the isolated bacteria C-11, S-22, L-17, and S-14 showed high identity with *B. velezensis*. The neighbor-joining tree showed that the isolates were classified into the cluster with *B. velezensis* (Supplementary Figure S4). Strain L-17 and S-14 had higher nucleotide sequence similarity value (85%) when compared to *B*. *velezensis* (GenBank accession numbers: KY630544, NC022530.1). The sequences were submitted to the GenBank database and the accession numbers of *B*. *velezensis* C-11, S-22, L-17, and S-14 were obtained (MN044920.1, MN044921.1, MN044922.1, MN044923.1). The *gyr*B (DNA gyrase B) and RNA polymerase beta subunit (*rpo*B) genes were used for further identification, which showed high similarity with *B. velezensis* OSY-GA1. The phylogenetic tree (Figure 1) suggested that the isolates formed a specific cluster with *B. velezensis*.

### Enzyme Production

**FIGURE 2:**
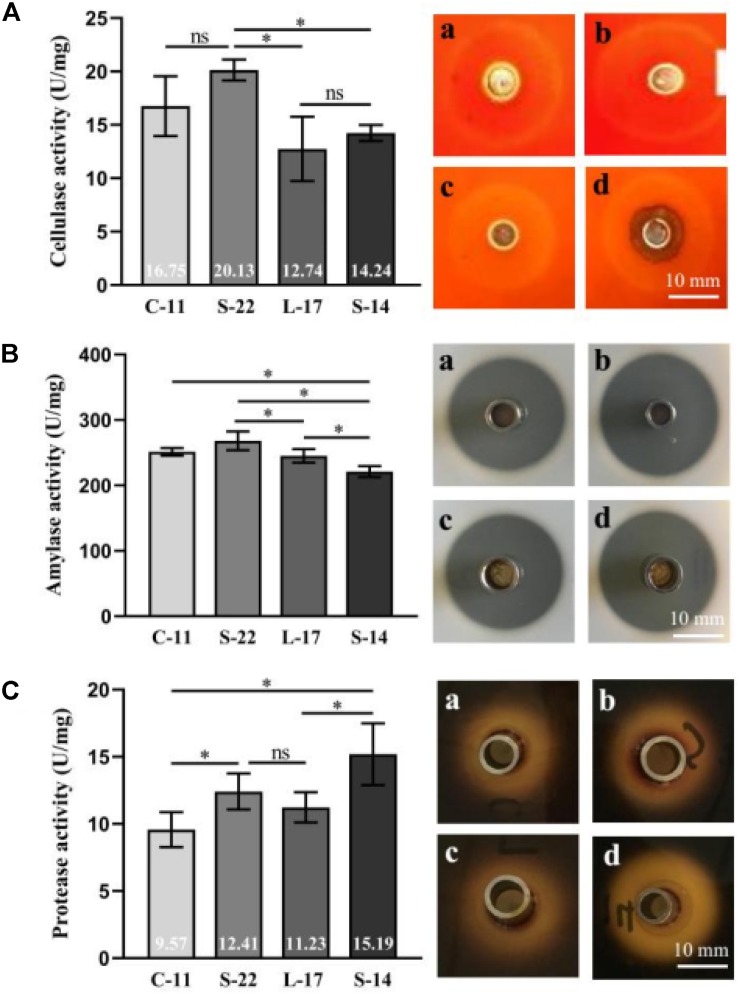
Characterization of cellulase **(A)**, amylase **(B)** and protease enzymes **(C)** in the presence of the *Bacillus velezensis* strains. (a) *B. velezensis* C-11 (b) *B. velezensis* S-22 (c) *B. velezensis* L-17 and (d) *B. velezensis* S-14. Data are presented as mean ± standard deviation (SD), and the results are representative of three independent experiments. Asterisks indicate statistical significance (*P* < 0.05), ns, not significant.

The cellulase, amylase, and protease activity of the isolates were examined by quantitative analysis and plate-based activity assays. The results showed that strain C-11, S-22, L-17, and S-14 have strong cellulase activity, which effectively degraded cellulose in an agar plate (Figure 2A). Moreover, the isolates were found to also possess amylase and protease activity (Figures 2B,C). Highest mean protease activity (15.19 U/mg) was exhibited by strain S-14 (Figure 2C). One way ANOVA followed by Tukey’s analysis showed that the protease activity of strain S-14 was significantly higher than the other bacterial isolates (*P* < 0.05).

### Tolerance of *Bacillus* Spores to Heat, Low pH, and Bile

**FIGURE 3:**
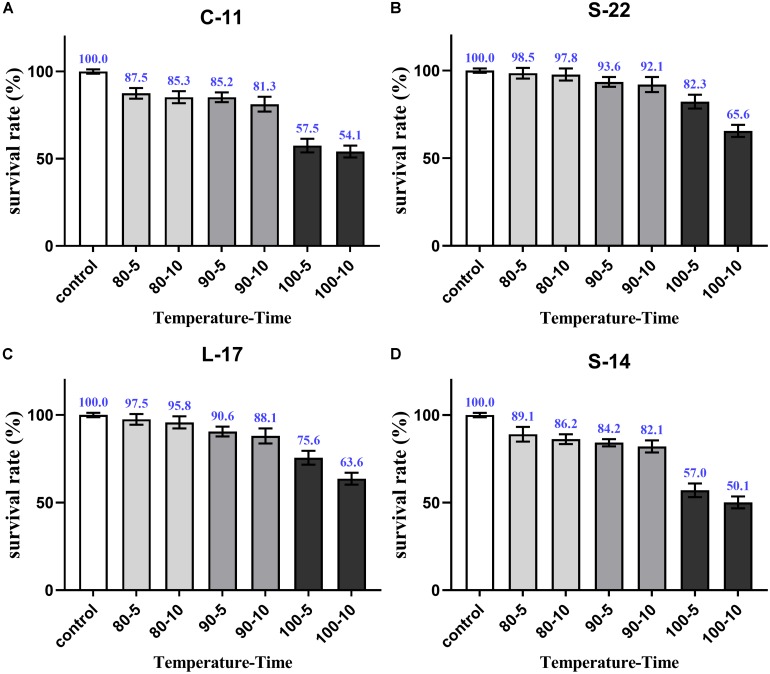
Heat resistance of the *B. velezensis* S-22 **(A)**, L-17 **(B)**, S-14 **(C)**, C-11 **(D)**. The survival rates of spores heated at 80, 90, and 100°C for 5 and 10 min separately are presented as means ± SD. The results are representative of three independent experiments.

As shown in Supplementary Figure S5, growth curve analysis showed that the *B. velezensis* isolates exhibited low growth during the early phase (1 h). The growth rate of the all isolates markedly increased during the logarithmic phase (from 1 to 2 h). As shown in Figure 3, high survival of the isolates against high temperature (100°C) was observed. The results showed that the strains S-22 and L-17 were more heat stable at 100°C for 5 min, with survival rates 82.3 and 75.6% (Figures 3A,B). While the rates of S-14 and C-11 reduced to about 57.0 and 57.5% (Figures 3C,D). Resistances to acid and bile salt conditions are tested to evaluate the tolerance of the spores and vegetative cells. At pH 3.0, the survival rates of the isolates were 96.03, 94.23, 95.12, and 97.23% respectively, and the viabilities reduced as pH decreased (Table 3). Furthermore, the high bile tolerance of the all spores was observed during the increase of bile salts concentrations from 2.5 to 10% (Table 4).

**TABLE 3:** Viabilities and survivability of the isolated probiotic at low pH.

**Strain**	**Viable count (log cfu/mL) and Survival rate (%)**			
	
	**pH**			
	
	**Control**	**3**	**2**	**1**
C-11	8.23 (100)	7.76 (96.03)	7.62 (91.12)	5.48 (76.14)
S-14	8.16 (100)	7.51 (94.23)	7.41 (88.41)	5.21 (73.24)
L-17	8.41 (100)	7.58 (95.12)	7.47 (89.24)	6.04 (80.21)
S-22	8.27 (100)	7.86 (97.23)	7.75 (94.23)	6.27 (83.12)

*Each value is the mean ± standard deviation (SD) of three independent experiments.*

**TABLE 4:** Viabilities and survivability of the isolated probiotic at different bile concentrations.

**Strain**	**Viable count (log cfu/mL) and Survival rate (%)**				
	
	**Bile (%)**				
	
	**Control**	**2.5**	**5.0**	**7.5**	**10.0**
C-11	8.03 (100)	7.76 (95.14)	7.56 (95.01)	7.21 (94.54)	7.15 (90.03)
S-14	8.16 (100)	7.51 (94.04)	7.43 (93.43)	7.24 (93.21)	7.17 (91.14)
L-17	8.11 (100)	7.58 (95.03)	7.50 (94.02)	7.17 (93.74)	7.03 (91.12)
S-22	8.17 (100)	7.86 (96.24)	7.69 (95.83)	7.47 (95.40)	7.29 (92.04)

*Each value is the mean ± standard deviation (SD) of three independent experiments.*

### Antibiotic Susceptibility of *Bacillus* Strains

All *B. velezensis* isolates were susceptible to Ampicillin, Piperacillin, Cephalexin, Cefazolin, Cefradine, Cefuroxim, Ceftazidime, Amikacin, Gentamicin, Kanamycin, Neomycin, Tetracycline, Doxycycline, Minocycline, Erythromycin, Norfloxacin, Ofloxacin, Ciprofloxacin, Furazolidone, Chloramphenicol, Amoxicillin, and Clindamycin. On the contrary, *B. velezensis* C-11 and S-22 showed resistance to Polymyxin B (Supplementary Table S3).

### Pathogenicity Test

The pathogenicity of the *B. velezensis* isolates for Crucian carp was evaluated through intraperitoneal injection and oral administration. No clinical signs of abnormalities were observed for the isolates against experimental fish compared to control. In addition, no impact on the swimming behavior, body weight, and mortality was found (data not shown). The histopathological analysis revealed that no damage was detected in the spleen, head kidney, liver, intestine, and heart of Crucian carp after oral administration with *B. velezensis* (Supplementary Figure S6).

### Phagocytic Assay

**FIGURE 4:**
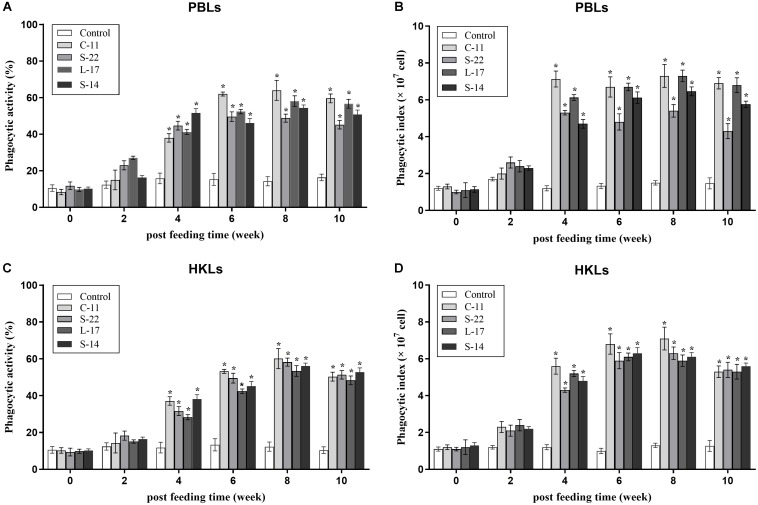
Phagocytic activities and phagocytic index of peripheral blood leukocytes (PBLs) **(A,B)** and head kidney leukocytes (HKLs) **(C,D)** in Crucian carp administrated with the *B. velezensis*. Data are presented as mean ± SD (*n* = 3). Asterisks indicate statistical significance compared with control (*P* < 0.05).

At 2 weeks after feed-trials, phagocytic activities of PBLs and HKLs were not statistical significance between the *B. velezensis* supplemented and control groups (*P* > 0.05). After 4 weeks, the fish treated with the probiotics exhibited significantly higher (*P* < 0.05) phagocytic activities compared to the control fish (Figure 4). At week 8, phagocytic activity (64.0%) of PBLs was enhanced significantly (*P* < 0.05) in *B. velezensis* C-11 fed group in comparison to the control group (Figures 4A,B). Similar result was also observed in HKLs of fish treated with *B. velezensis* C-11 (60.1%) at week 8 (Figures 4C,D).

### Non-specific Immune Parameters

**FIGURE 5:**
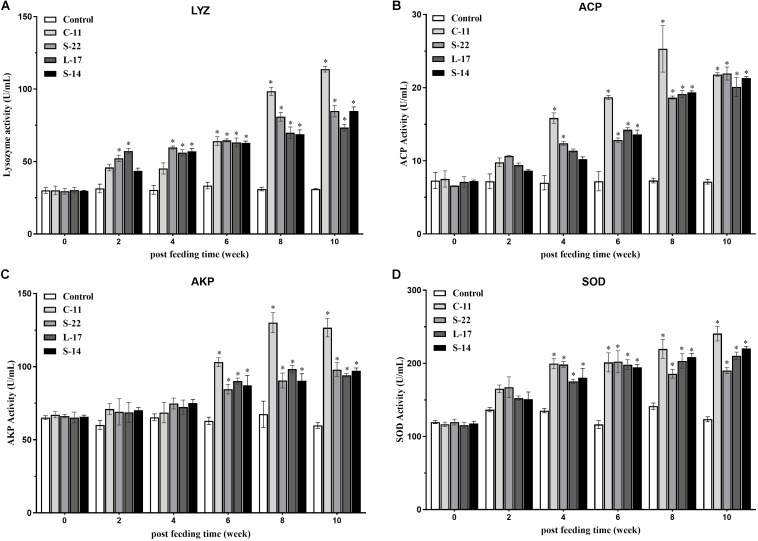
**(A)** Serum lysozyme (LYZ) activity, **(B)** acid phosphatase (ACP) activity, **(C)** alkaline phosphatase (AKP) activity, and **(D)** superoxide dismutase (SOD) activity after supplementation of Crucian carp with different *B. velezensis* probiotics diets. Values are presented as mean ± SD (*n* = 3). Asterisks indicate statistical significance compared with control (*P* < 0.05).

**FIGURE 6:**
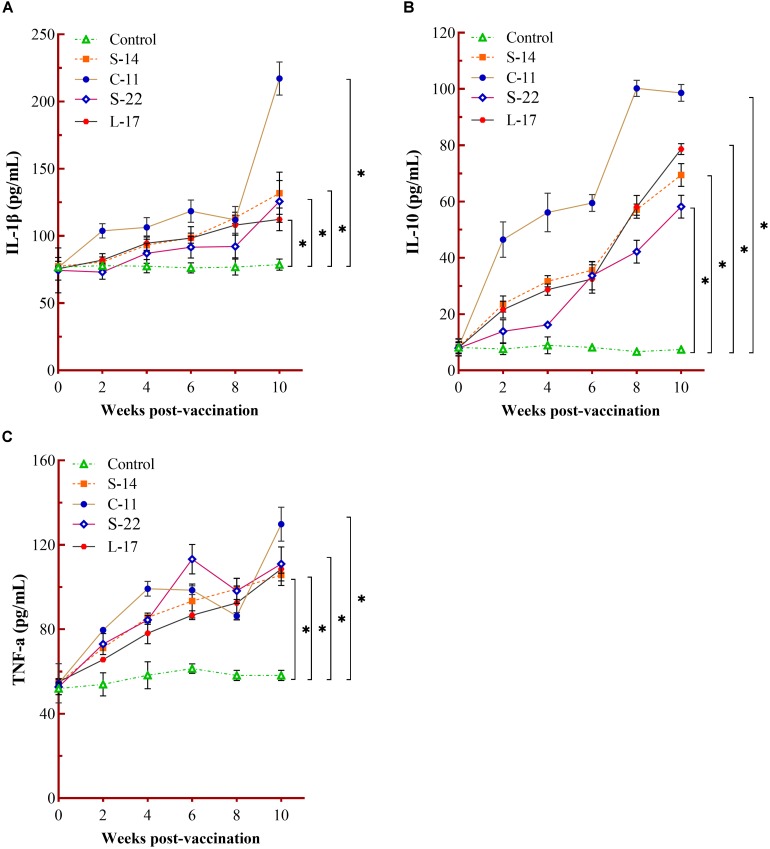
Measurement of cytokines activity in serum. Serum IL-1β **(A)**, IL-10 **(B),** and TNF-α **(C)** activity after being fed with different *B. velezensis* probiotics diets for 10 weeks. Each value represents mean ± SD (*n* = 3). Asterisks indicate statistical significance compared with control (*P* < 0.05).

**FIGURE 7:**
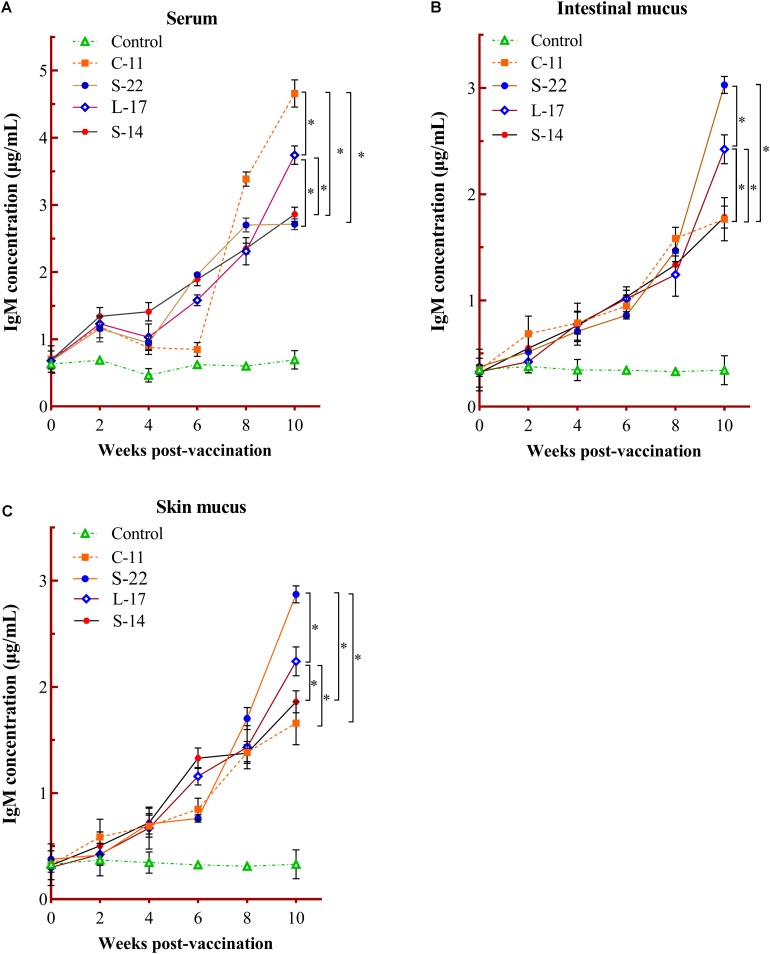
Serum **(A)**, intestinal mucus **(B),** and skin mucus **(C)** IgM responses triggered by oral administration of the different *B. velezensis* probiotics diets. Values are presented as mean ± SD (*n* = 3). Asterisks indicate statistical significance compared with control (*P* < 0.05).

The humoral immune parameters of Crucian carp treated with diets containing *B. velezensis* were determined. During feed-trials period, the serum lysozyme activity of fish received *B. velezensis* C-11 had significantly (*P* < 0.05) higher activity compared to control group at week 8 (98.45 U/mL) and week 10 (113.76 U/mL) (Figure 5A). A significantly increased ACP activity was observed in *B. velezensis* C-11 and S-22 groups as compared with control group after 4 weeks (*P* < 0.05, Figure 5B). Similarly, fish exhibited significantly higher AKP activity in all treatment groups after 4 week of administration (*P* < 0.05, Figure 5C). Moreover, the levels of SOD activity in probiotic groups were significantly higher after second week of feeding (*P* < 0.05, Figure 5D), and the *B. velezensis* C-11 group had a higher SOD activity among all the probiotic groups (Figure 5D). Among the measured cytokines in serum, IL-1β, IL-10, and TNF-α activity of fish treated with *B. velezensis* C-11 showed higher significant values (*P* < 0.05, Figure 6). After 6 weeks, all the probiotic groups displayed higher activity of IL-1β, IL-10, and TNF-α compared with the control group (Figure 6). The evaluation of IgM in serum, intestinal mucus, and skin mucus from fish fed with probiotics was performed. Significant higher levels of IgM were observed in serum, intestinal mucus and skin mucus samples in different *B. velezensis* groups since week 6, when compared with the control group (*P* < 0.05, Figure 7). Levels of IgM in samples of serum, intestinal mucus, and skin mucus in probiotic groups kept rising till week 8, and maintained significantly high levels until 10 weeks (Figure 7). Significant higher levels of IgM were observed in serum samples of groups of *B. velezensis* C-11 and L-17 at 10 week post-vaccination, when compared with *B. velezensis* S-22 and S-14 groups (*P* < 0.05, Figure 7A). However, the level of IgM in serum did not differ between *B. velezensis* S-22 and S-14 groups (*P* > 0.05, Figure 7A). Levels of IgM in samples of intestinal mucus in *B. velezensis* S-22 and L-17 groups maintained significantly high levels throughout experiment compared to *B. velezensis* C-11 and S-14 groups (*P* < 0.05, Figure 7B) at 10 week post-feeding, whereas no statistical differences were observed between *B. velezensis* C-11 and S-14 groups. Similarly, the levels of IgM in samples of skin mucus in the *B. velezensis* S-22-treated group increased significantly at 10 week post-vaccination compared with *B. velezensis* L-17, C-11, and S-14 groups (*P* < 0.05, Figure 7C). Meanwhile, a slow increase of IgM level in the *B. velezensis* C-11 and S-14 groups occurred after 8 week post-administration (Figure 7C).

### Quantification of Spores in Intestine Tract

The intestines in probiotic groups were collected and quantify the presence of *B. velezensis* strains in the whole Crucian carp gastrointestinal tract. As shown in Table 5, the spores were not detected in the gut samples of groups before the trial. At weeks 6, 8 and 10, however, the *B. velezensis* isolates were determined in the fish intestine samples of the probiotics treatment groups (Table 5). No strains were found in the control group during the administration.

**TABLE 5:** Quantification of total *Bacillus velezensis* strains in the intestine of Crucian carp following qRT-PCR analysis.

**Treatments**	**Week 0, (CFU/g)**	**Week 6, (CFU/g)**	**Week 8, (CFU/g)**	**Week 10, (CFU/g)**
Control	ND	ND	ND	ND
*B. velezensis* C-11	ND	0.93 ± 0.2 × 10^6^	1.69 ± 0.1 × 10^7^	1.18 ± 0.2 × 10^5^
*B. velezensis* S-22	ND	1.14 ± 0.1 × 10^4^	0.79 ± 0.3 × 10^6^	1.32 ± 0.2 × 10^5^
*B. velezensis* L-17	ND	1.09 ± 0.4 × 10^5^	1.31 ± 0.1 × 10^6^	0.72 ± 0.1 × 10^4^
*B. velezensis* S-14	ND	1.24 ± 0.3 × 10^6^	1.53 ± 0.2 × 10^6^	1.02 ± 0.3 × 10^5^

*Values are presented as mean ± SD (*n* = 3). ND, not determined.*

### Immune Gene Expression

**FIGURE 8:**
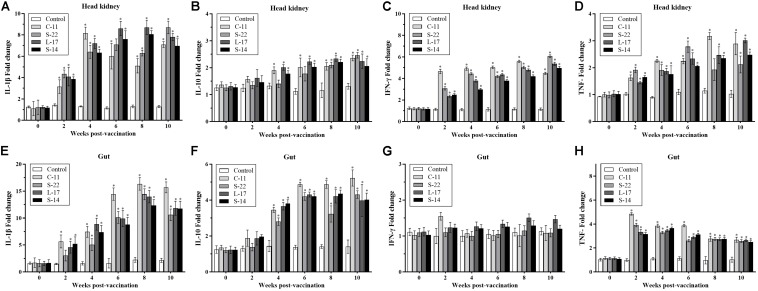
qRT-PCR analysis of the expression of **(A,E)** interleukin-1β (IL-1β), **(B,F)** interleukin-10 (IL-10), **(C,G)** interferon gamma (IFN-γ), and **(D,H)** tumor necrosis factor-α (TNF-α) gene in head kidney and gut of Crucian carp (*n* = 3 fish/group) after administration. Data are presented as mean ± SD (*n* = 3). Asterisks indicate statistical significance compared with control (*P* < 0.05).

Expression of the pro-inflammatory cytokines in intestine and head kidney of Crucian carp was examined. As shown in Figure 8, the *B. velezensis* strains C-11, S-22, L-17, and S-14 induced similar expression patterns of the tested cytokines. Our data showed that the mRNA level of cytokine interleukin 1β (IL-1β) was up-regulated until 8 week, and *B. velezensis* restrained up-regulation of IL-1β in head kidney and intestine tissues after 8 week (Figures 8A,E) compared to the control group. The increased IL-10 transcripts were obtained in fish fed diets containing *B. velezensis* throughout the 10-week study period (*P* < 0.05, Figures 8B,F). A higher IFN-γ expression was observed after 2 weeks of feeding (*P* < 0.05) in head kidney compared to control group, while an initial level of IFN-γ maintained in intestine during the 10-week trial (Figures 8C,G).

In addition, compared with control, the mRNA expression of TNF-α was significantly higher in *B. velezensis* feeding groups after 2 weeks of feeding in head kidney (*P* < 0.05, Figure 8D). In contrast, down-regulation of TNF-α was observed in gut samples after 4 weeks of administration (Figure 8H).

### Histological Analysis

**FIGURE 9:**
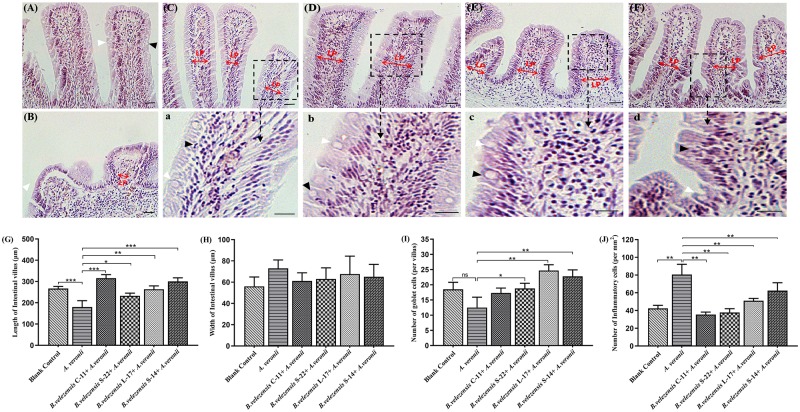
Hematoxylin-eosin staining of gut cryosections from blank control **(A)**, *A. veronii* **(B)**, *B. velezensis* C-11 **(C)**, *B. velezensis* S-22 **(D)**, *B. velezensis* L-17 **(E),** and *B. velezensis* S-14 **(F)** after challenge with *A. veronii*. In each panel, white arrows indicate goblet cells; black arrows indicate inflammatory cells. Lamina propria (LP) is shown (Scale bars: 20 μm). (a–d) Enlarged images of the areas outlined in **(C–F)**. **(G)** The lengths of intestinal villi in the mid-intestine. **(H)** The widths of intestinal villi in the mid-intestine. **(I)** The number of goblet cells in intestinal villi. **(J)** The numbers of inflammatory cells in intestinal villi. ^∗^*P* < 0.05, ^∗∗^*P* < 0.01, and ^∗∗∗^*P* < 0.001. Data are representative of three independent experiments.

Compared with the control group, histological examination of the mid-intestine of Crucian carp fed the probiotic diet for 8 weeks demonstrated intestine inflammation injury was decreased after challenge with *A. veronii* (Figure 9). Additionally, the growth of intestinal villi and increasing the numbers of goblet cell could be promoted by *B. velezensis*. Few inflammatory cells infiltrated the lamina propria (LP) and intestinal mucosa in the intestines of control group (Figures 9A,J). In the *A. veronii* group, as shown in Figure 9J and Supplementary Table S4, *A. veronii* induced infiltration of a large number of inflammatory cells into the mucosa when compared to control fish (*P* < 0.01), and shedding and swelling was observed in the intestinal villi (Figure 9B). Although inflammatory cells infiltrated the LP and mucosa was detected in the *B. velezensis* + *A. veronii* groups, intestinal villi were intact and a notable goblet cells accumulation as compared to *A. veronii* group (*P* < 0.05, Figure 9 and Supplementary Table S4). Moreover, the length of the intestinal villi significantly decreased, and the width of intestinal villi increased in the *A. veronii* group (*P* < 0.05, Figures 9G,H). However, no significant difference in intestinal villi length and width was observed between control and *B. velezensis* + *A. veronii* groups (Supplementary Table S4).

### Evaluation of Protective Efficacy

**FIGURE 10:**
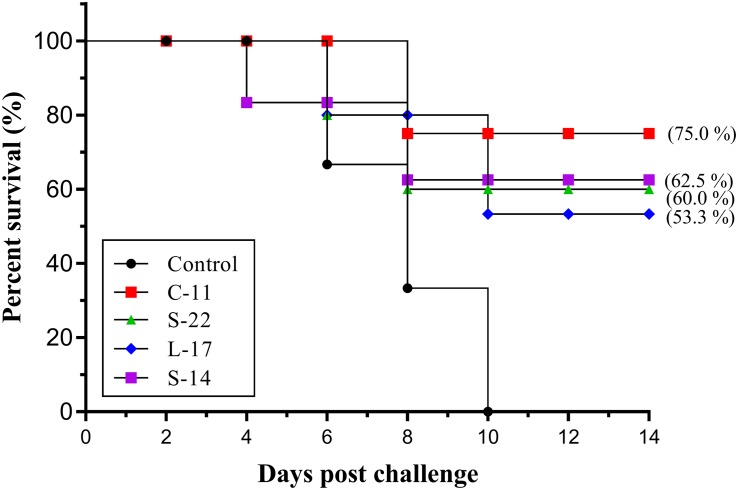
Survival rate of fish oral administration with *B. velezensis* C-11, *B. velezensis* S-22, *B. velezensis* L-17, and *B. velezensis* S-14 or PBS following challenge with the *A. veronii* TH0426 strain. Thirsty fish of each group were used to record percent survival for 14 day.

Two weeks post *A. veronii* challenge, the protective effect of the *B. velezensis* on Crucian carp against bacterial infection was determined as shown in Figure 10. A significant increase in the mortality of the control group (100%) was detected during the infection. Conversely, the survival rates in the fish orally immunized probiotic groups (75.0, 62.5, 60.0, 53.3%) were higher compared to the control group after 14 days post-infection (Figure 10). All dead fish exhibited severe hemorrhage and blood congestion in the liver and spleen, and gut displayed an accumulation of yellowish liquid. Pure bacterial colonies resembling *Aeromonas* spp. were recovered from the liver, kidney and spleen of the dead fish, as confirmed by colonial morphology observation and molecular identification (data not shown).

## Discussion

The fish gastrointestinal tract houses diverse microorganisms, and studies on gut microbiota have been reported in gibel carp (*Carassius auratus* gibelio), Atlantic salmon (*Salmo salar*) and Aangasius catfish (*Pangasius bocourti*) (Wu et al., 2013; Gajardo et al., 2016; Meidong et al., 2017b). Currently, the gut microbiota has been reported for other probiotics such as *Bacillus* spp. and lactic acid bacteria, which proposed to constitute an alternative treatment for antibiotics associated adverse effects in aquaculture (Kazun′ and Kazun′, 2013). However, few studies have reported the mechanism of damage caused by *A. veronii* and the protective effects of probiotics on the intestinal mucosal barrier in fish. In this study, we found that the potential probiotics *B. velezensis* C-11, S-22, L-17, and S-14 isolated from gut of healthy Crucian carp, displayed positive probiotic properties on antimicrobial activity against fish pathogens (*A. veronii* and *A. hydrophila*), resistance to gastrointestinal tract conditions and production of digestive enzyme (cellulase, amylase, and protease). Hence, we decided to investigate further the capacity of *B. velezensis* to regulate immune responses in fish, using the Crucian carp as a model.

Phylogenetic analysis of the 16S rRNA gene sequence is hardly distinguishable for bacterial classification at species level due to highly conserved nature of this gene (Rooney et al., 2009). The *gyr*B and *rpo*B genes have been used as representative markers for accurate identification of *Bacillus* (Chun and Bae, 2000). The results confirmed that the bacteria isolated from Crucian carp gut were *B. velezensis* by microbial properties and molecular identification (Figure 1). The outbreaks of bacteria disease cause severe economic losses to aquaculture industry, which leads to antibiotics widespread employed in fish farming (Bentzon-Tilia et al., 2016). *Bacillus* spp. isolated from healthy fish gastrointestinal tract have been widely employed as natural alternatives to antibiotics and chemical treatments for the control of diseases (Lee et al., 2016; Rauta et al., 2016; Meidong et al., 2017a). Antimicrobial activity is recognized one of the major mechanisms through which probiotics function (Alleson et al., 2012). This study revealed that *B. velezensis* C-11, S-22, L-17, and S-14 displayed strongly antagonistic activity against fish pathogens (*A. veronii* and *A. hydrophila*). Interestingly, 3 g-positive pathogens (*S. aureus*, *S. agalactiae*, and *C. perfringens*) also effectively inhibited by all strains of *B. velezensis* (Supplementary Table S2), indicating that antagonistic property of broad spectrum of *Bacillus* isolates could be applied against genera *Aeromonas* and *Streptococcus* in aquaculture. Meidong et al. (2017a) studied the disease resistance and probiotic effects of *Bacillus siamensis* isolated from pickled vegetables, their results demonstrated that strains exhibited a broad-spectrum antibacterial activity (*A. hydrophila* and *S. agalactiae*) and conferred significant benefit in reducing catfish mortality when challenged by *A. hydrophila*. These observations implied that production of antimicrobial compounds and biofilm formation by *Bacillus* strains involved the antagonistic mechanisms (Aleti et al., 2016).

It has been reported that ability to produce hydrolytic enzymes enhances the digestion of macromolecules in the gastrointestinal tract of fish and increase degradation of digesta (Ray et al., 2012). Our study highlighted the three main enzyme activities produced by *Bacillus* strains possible contribute to food digestion. The enzyme activity measurement revealed that cellulase and amylase activity of *B. velezensis* C-11 and S-22 was significantly higher than (*P* < 0.05) than strains L-17 and S-14, while protease activity of strain S-14 was much higher than others isolates (Figure 2). This result is similarly related to the enzyme activity of *B. subtilis*, clearly demonstrated by Guo et al. (2016) correspondence analysis. Although whether the hydrolytic enzymes are produced by the certain gut microbiota or host is not exactly know in fish, our study indicated that *B. velezensis* isolated from fish may influenced enzyme activities of gut microbiota. Yokoe and Yasumasu (1964) mentioned that endogenous cellulose was absent in gut content and alimentary tract of Crucian carp (*Carassius auratus*) and Common carp (*Cyprinus carpio*). However, a previous study determined the cellulase activity in intestine and hepatopancreas of Rohu (*Labeo rohita*), and confirmed that the gut cellulase activity largely contributed by the gut microbiota (Saha and Ray, 1998). These researches indicated that certain gut microbiota might be good candidates as probiotic to improve food utilization efficiency in aquaculture.

The ability of the *Bacillus* isolates to survive and grow at high temperature and tolerance to acid and bile are the foundation for probiotics. Isolates identified in the present study were not sensitive to 80 and 90°C, and survived even up to 100°C. As expected, the spores were found to exhibit tolerance to acidity and high bile concentration (Tables 3, 4), which demonstrates the isolates could survive acid and bile condition in the intestine. Similar to our study, *B. subtilis* isolated from the intestine of the grass carp as probiotic with tolerances to gastric and intestinal conditions (Guo et al., 2016). To provide evidence that potential aquatic probiotic of *B. velezensis* isolates, the strains were intraperitoneally injected and orally administrated. Our data confirmed that the *B. velezensis* isolates were safe to Crucian carp. In addition, *B. velezensis* isolates are susceptible to most of the common antibiotics tested, which indicated that the isolates have inability to induce multidrug resistance and transfer antibiotic-resistant genes to organism of host gut.

As Nile tilapia (*Oreochromis niloticus*) IL-1β and TNF-α induced the recruitment of phagocytic cells in gut (Galagarza et al., 2018) and taking into account that B cells in fish have phagocytic and microbicidal properties associated to their phagocytic activity (Li et al., 2006), we also studied the effect of the probiotic on phagocytic activity of Crucian carp B cells. In our experiments, the fish fed diets containing *B. velezensis* isolates showed significantly higher phagocytosis of PBLs and HKLs than the control fish after 8 weeks of administration (Figure 4). Additionally, the phagocytic activity in HKLs appeared to be enhancement, which indicates that the head kidney being an immune organ and predominantly constituted by potent phagocytes in teleosts. As a consequence of immunity activation, the up-regulation of different phagocytic cells recruitment should be observed. However, phagocytic activities of B cells were not statistical significance between the probiotic and control groups at week 2, it seems possible that the *B. velezensis* are modulating phagocytic cells activation by colonization, and this should be studied further. Nevertheless, these observations highlight the potent antimicrobial characteristics of fish B cells.

Increased levels of cytokines induce inflammation and trigger phagocytic cells at the infection site, which keep the dynamic balance of host immune response. In fish, many studies have indicated that regulation in T cells and B cells were mediated by cytokines such as TNF-α and IL-1β, or infection by pathogens (Plazadiaz et al., 2014; Wu et al., 2015). Meanwhile, *in vivo* studies have shown that pretreatment with probiotics can protect Crucian carp and Nile tilapia from *A. hydrophila* (Hamdan et al., 2016; Yi et al., 2018). Park et al. (2010) demonstrated that *B. subtilis* secreting surfactin could regulate inflammatory response by reducing the expression of pro-inflammatory cytokines through interruption of signal pathway (nuclear factor-kappa B, NF-κB). Our results confirmed that *B. velezensis* isolates induced immune responses and the expression levels of immune-related genes (IL-1β, IFN-γ, and TNF-α) were up-regulated in gut and head kidney of Crucian carp at earlier stage of feeding (Figure 8). Besides, the probiotics also increased the serum level of IL-1β, IL-10, and TNF-α in serum (Figure 6). We speculated that *B. velezensis* probiotics applied in this study may enhance the appropriate commensal bacterial colonization in gut, which might trigger activation of lymphocytes, macrophages and natural killer cells (NK cells) that release a variety of pro-inflammatory mediators. Dietary supplementation with probiotics improved immune responses in serum and tissues of fish, as evidenced in previous study with the use *B. amyloliquefaciens* in *L. rohita* (Nandi et al., 2017). Similar results were reported by Yi et al. (2018) found that *B. velezensis* could activate immune response, which results in an increase of the mRNA expression of IL-10 and IL-4 in the head kidney of *C. auratus*. That is to say, *B. velezensis* altered immune function, which may be attributed to distribution of the cytokines. Of note, *B. velezensis* entered into gastrointestinal tract might play a major role in modulation or homeostasis of fish, which could improve the pathogens or opportunistic pathogens clearance by means of *B. velezensis* carried strongly antagonistic activity against fish pathogens. It is interesting to note that *B. velezensis* effectively increased TNF-α mRNA expression levels of gut 2 weeks after administration and then down-regulated the expression of TNF-α after 4 weeks. The different modulation effects of probiotics supplementation on TNF-α activity may be related with the bioactivity of the cytokine. We inferred that the pro-inflammatory cytokines may be secreted through the accumulation of macrophage or monocytes in intestine, which could increase the permeability of the intestinal epithelium (Amasheh et al., 2009), the distinct mechanisms merit further research.

The plasma lysozyme, ACP, AKP, and SOD concentration are considered as innate immune parameters following dietary supplementation of probiotics (Magnadóttir, 2006). As seen in our study, the increased serum lysozyme, ACP, AKP, and SOD activities (*P* < 0.05) were obtained in fish stimulated with the probiotics throughout the 10-week study period. Similarly, Timothy and Dibyendu (2015) reported that catla (*Catla catla*) fed with *B. subtilis* supplemented diets showed higher levels of lysozyme and AKP compared to the control group. In rohu, a significant elevation of the serum lysozyme activity was observed in fish fed with diets containing *B. licheniformis* and *B. pumilus* for 2 weeks (Ramesh et al., 2015). By contrast, the serum lysozyme activity of tilapia treated with *Bacillus* strains showed no difference among the treatment groups after 40 days trial (Zhou et al., 2010). Thus, it seems that the non-specific defense mechanism generated in response to antigens may not be exclusively specialized in humoral component secretion, and plays a key role in pathogen clearance or antigen presentation, and in line with this hypothesis, the probiotics used in this study provoked significant phagocytic activities in the PBLs and HKLs.

In the absence of lymph nodes, teleost skin, and gastrointestinal tract constitutes as the main physical and immunological barriers, which involved in the immunity against pathogenic invasion and immune surveillance (Salinas et al., 2011). In teleost fish, specific secretory immunoglobulins (Igs) are found in lymphoid organs (head kidney and spleen), liver, circulating blood and mucosal surfaces (gut and skin) (Pignatelli et al., 2014). Three classes of Igs have been identified in teleosts (IgM, IgD, and IgT or IgZ), with IgM being the prevalent form and well-characterized Ig isotype in serum and mucosa-associated lymphoid tissues (MALTs) (Magadan et al., 2015; Jenberie et al., 2018). Our results demonstrated that IgM could be significantly triggered in serum, intestinal mucus and skin mucus of Crucian carp following administrated with *B. velezensis* diets. Compared with control groups, *B. velezensis* C-11 and S-22 diet evoked stronger systemic and mucosal immune responses (Figure 7). In agreement with our results, one study in grass carp has confirmed that specific immune responses with higher levels of IgM in serum and mucus samples induced by engineered *B. subtilis* (Tang et al., 2017). These observations indicated that fish IgM^+^ B cells exhibit both innate and adaptive immune functions and play a key role following vaccination (Ye et al., 2013), but the characteristics of distinct B cell subsets remain unknown.

Resistance to extreme gastrointestinal tract conditions by multiple layers structure of spores make it possible to develop an effective oral delivery vehicle (Zhenwen et al., 2015). Our data showed that *B. velezensis* spores could inhabit the gut of Crucian carp (Table 5), which confirmed that the ability of *Bacillus* strains to reach to the fish lumen successfully and propagate as part of the Crucian carp gut microbiota. Previously study has reported that *B. subtilis* spores could colonize in the foreguts and hindguts of grass carp, and maintained immunogenicity of protein expressed on the bacterial surface through the gastrointestinal tract (Tang et al., 2017). Disruption of intestinal barrier functions is related to intestinal inflammation, which causes translocation of bacterial antigens and induces expression of pro-inflammation cytokines (Turner, 2006). Probiotics exhibit antagonistic effects to prevent inflammation through competitive adherence to epithelium or mucus (Monica and Warren, 2007). Given this probiotics effect, it would be important to further investigate probiotics-induced factors contributing to the inhibition of inflammation and maintenance of gut immune homeostasis. A large number of intraepithelial lymphocytes infiltrated the epithelial cells of intestinal villi and significantly promote the growth of intestinal villi after treated with *B. subtilis* (Tang et al., 2017). Of particular note, the similar results obtained in this study indicated that few inflammatory cells infiltrated the mucosa in the *B. velezensis* + *A. veronii* groups, and numerous goblet cells accumulated in the intestinal villi (Figure 9). Research verified that supplementation *Lactobacillus rhamnosus* could effectively promote intestinal health by increasing the population of intraepithelial lymphocytes and the villous height in tilapia model (Pirarat et al., 2011; Sewaka et al., 2019).

The intestinal tract have been considered the primary probiotics colonization site, there is increasing evidence that diseases prevented and controlled using probiotics in aquaculture (Saputra et al., 2016). Dietary administration of *B. velezensis* for 8 weeks yielded significantly higher survival rates compared to the control group challenged by *A. veronii* (Figure 10). *B. amyloliquefaciens* supplemented-diet showed protection against *A. hydrophila* in Nile tilapia (Saputra et al., 2016), in line with our observations with *in vivo* probiotic administration. Similar results were observed in *L. rohita* treated with *L. plantarum* supplemented-diets and infected with *A. hydrophila* (Giri et al., 2013). Synthesis of mammals, fish and *in vitro* data points to an antagonistic activity of probiotics species, potentially mediated by their previously produced anti-microbial substance (Caballero et al., 2017). It appears likely that live probiotic obtained with animals could reduce intestinal colonization by pathogens, and susceptibility to infection, such interference may be reached by blocking a signaling system of pathogen. Whether host intestine occur with other probiotics not determined in our study, merit further studies.

Scientific evidence to support the statements that probiotic improve disease resistance and health in fish is scarce. However, this study provides evidence for mechanism underlying damage to *A. veronii* infections by which probiotic bacteria found in fish could regulate the local mucosal immune response and systemic immune response. Notably, we found the responsible agents to work by anti-microbial activity, demonstrating that pathogen exclusion in the intestine may work by inhibition of pathogen colonization. Furthermore, our findings emphasize the importance of *B. velezensis* for immunomodulatory. Our study suggests a valuable application regarding alternative strategies to replace antibiotics, and *Bacillus*-containing dietary could be used for safe *A. veronii* decolonization strategy. Such a probiotic approach would have numerous benefits over the typical method involving antibiotics, which is aimed effectively at inhibiting the colonization of pathogen.

## Data Availability Statement

The datasets generated for this study have been deposited at GenBank under the accession numbers MN044920.1, MN044921.1, MN044922.1, and MN044923.1. The datasets cited for previous sequencing data can be found in GenBank under the accession numbers KY630544 and NC022530.1.

## Ethics Statement

The animal study was reviewed and approved by the Jilin Agriculture University Institutional Animal Care and Use Committee (JLAU08201409) and carried out in accordance with the National Institutes of Health Guide for the Care and Use of Laboratory Animals (NIH Publications No. 8023).

## Author Contributions

All authors wrote the manuscript, designed and performed the experiments, read, and commented on the manuscript. D-XZ, and Y-HK carried out the statistical analyses and organized the data. SZ and Z-LZ created the figures. X-FS and A-DQ supervised the research design and revised the manuscript.

## Conflict of Interest

The authors declare that the research was conducted in the absence of any commercial or financial relationships that could be construed as a potential conflict of interest.
