# Supplementary material for: Effect of Bacillus velezensis on Aeromonas veronii-Induced Intestinal Mucosal Barrier Function Damage and Inflammation in Crucian Carp (Carassius auratus)
Source: Front Microbiol. 2019 Nov 15;10:2663. doi: 10.3389/fmicb.2019.02663 (PMC6874145; doi:10.3389/fmicb.2019.02663)
Supplement: Supplementary file 1 [file Data_Sheet_1.doc]

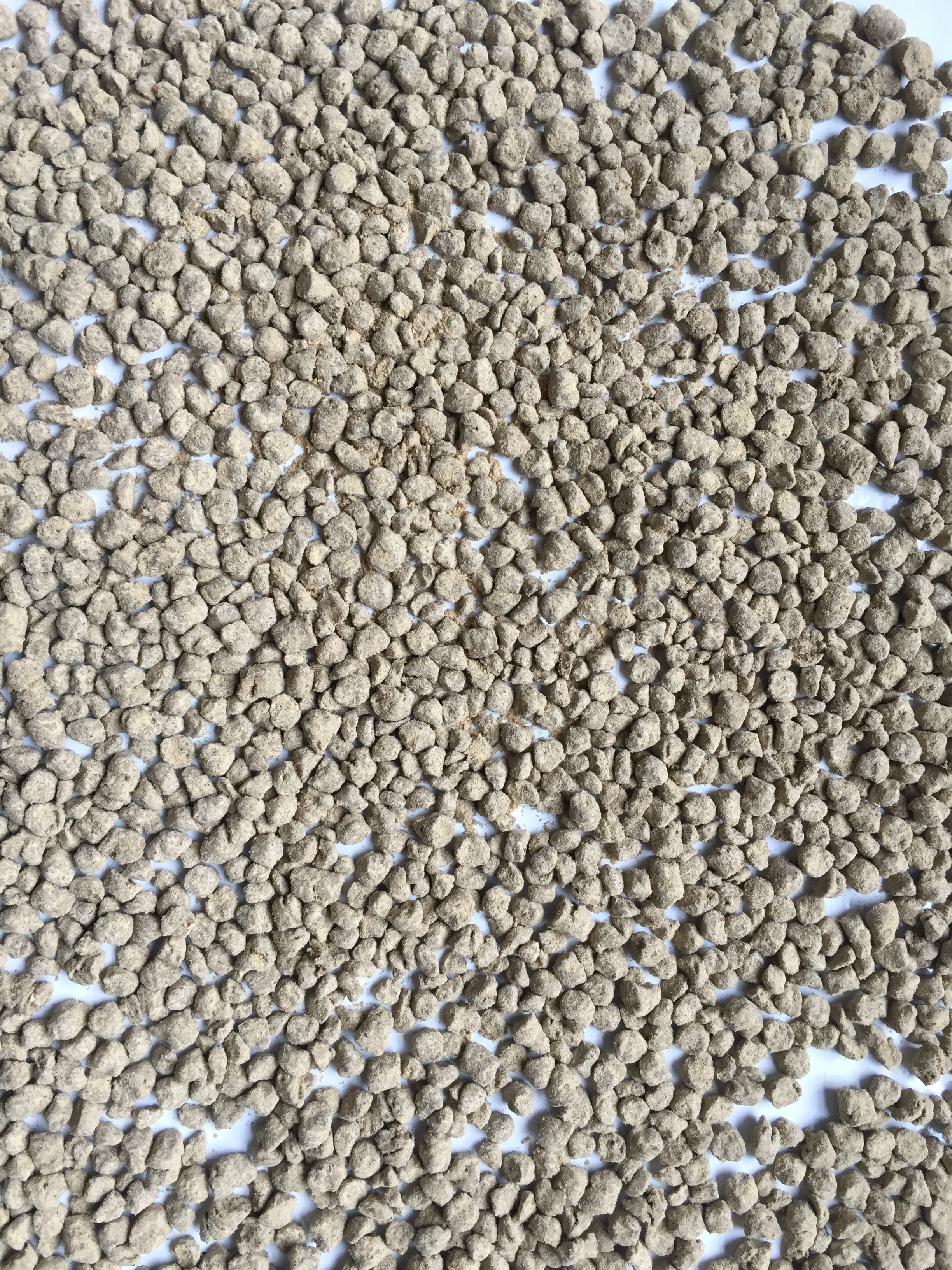


1 cm

**Fig.S1**. Bait particles containing *Bacillus* used for oral administration. Overnight cultures of *Bacillus* strains were fully mixed with basal diet feed, and were oven-dried at 40 ℃ for 6 h and containing 10^8^ colony forming unit (CFU)/g of the probiotic on average.

**(A)**


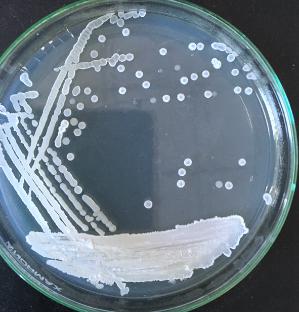

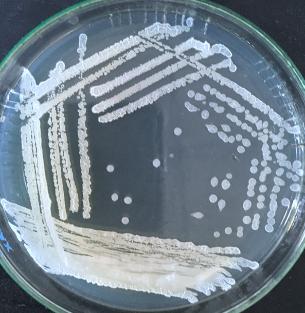


**a**

**b**


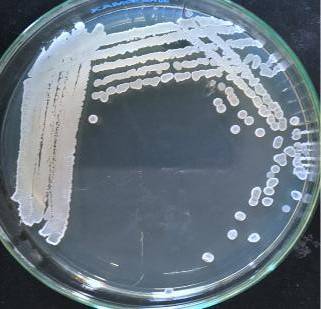


**c**


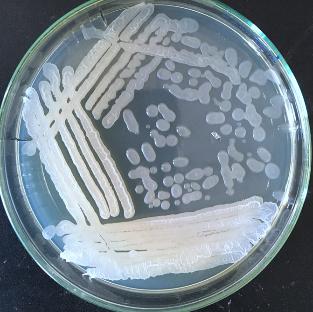


**d**


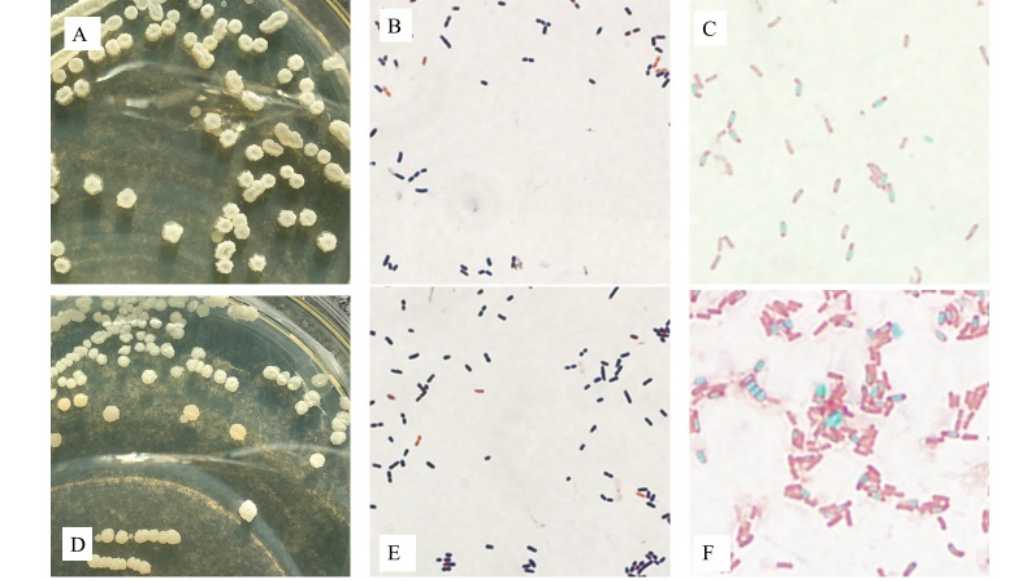

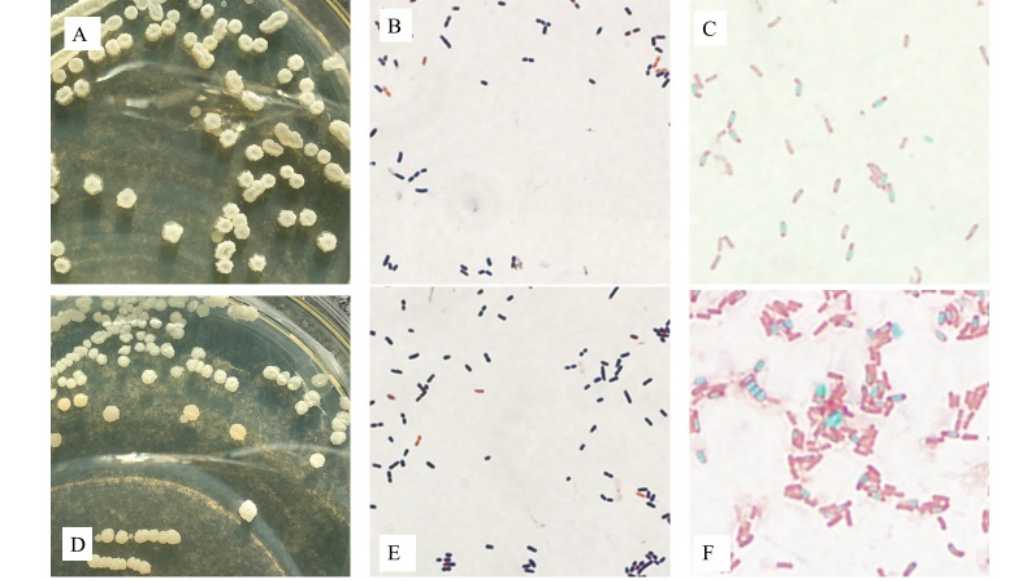


**a**

**b**


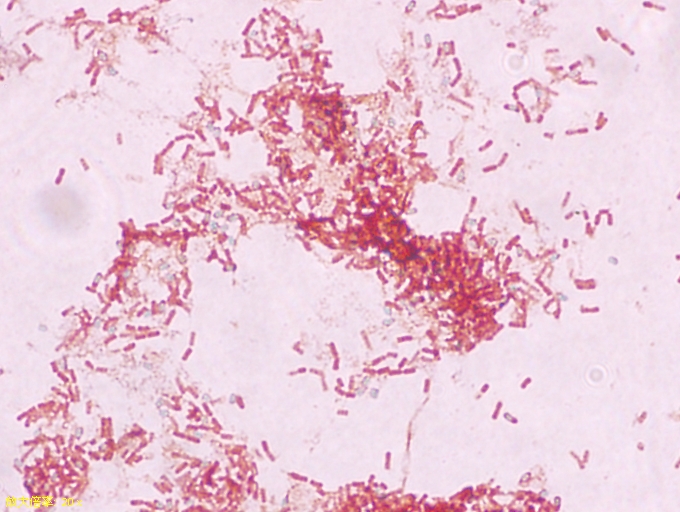

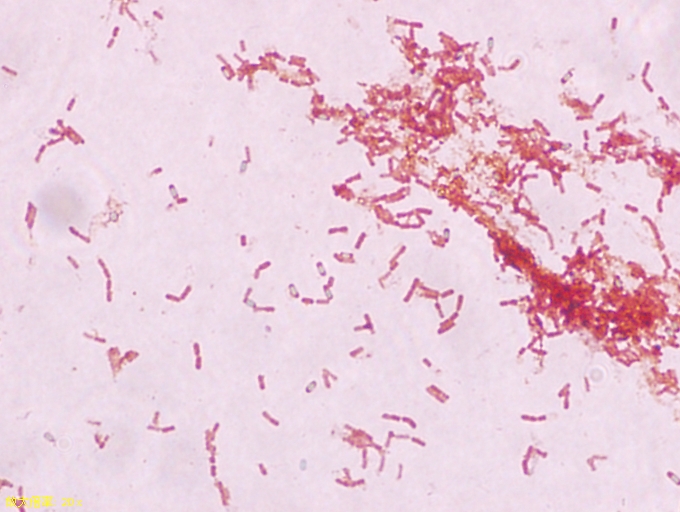


**c**

**d**

**(C)**

1000 ×

**(B)**


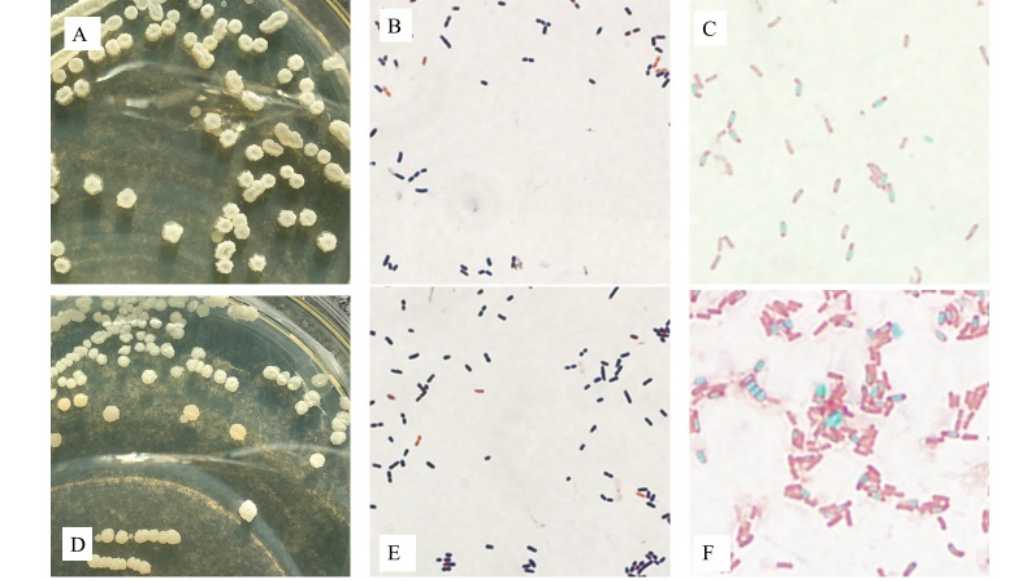

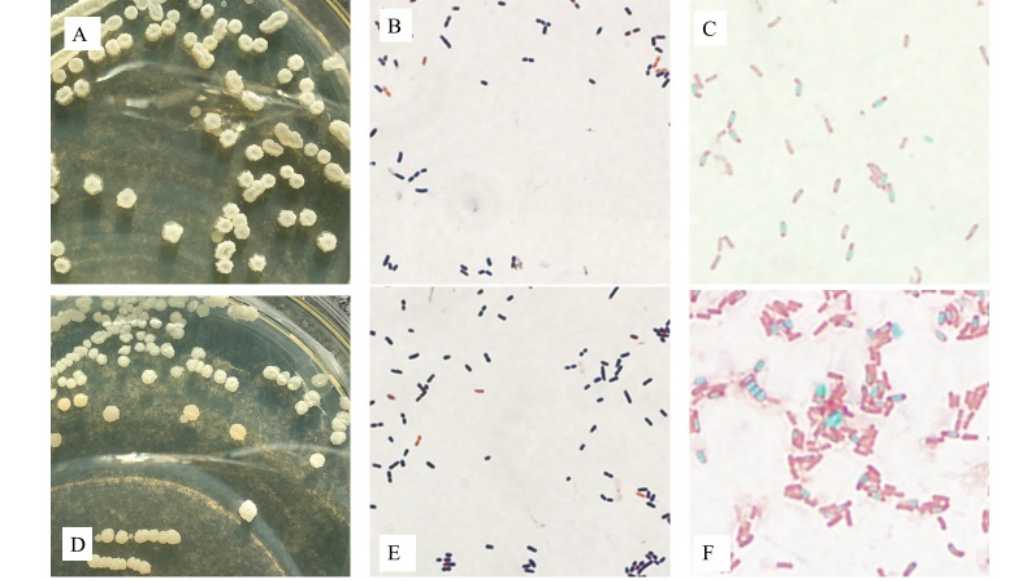


**a**

**b**


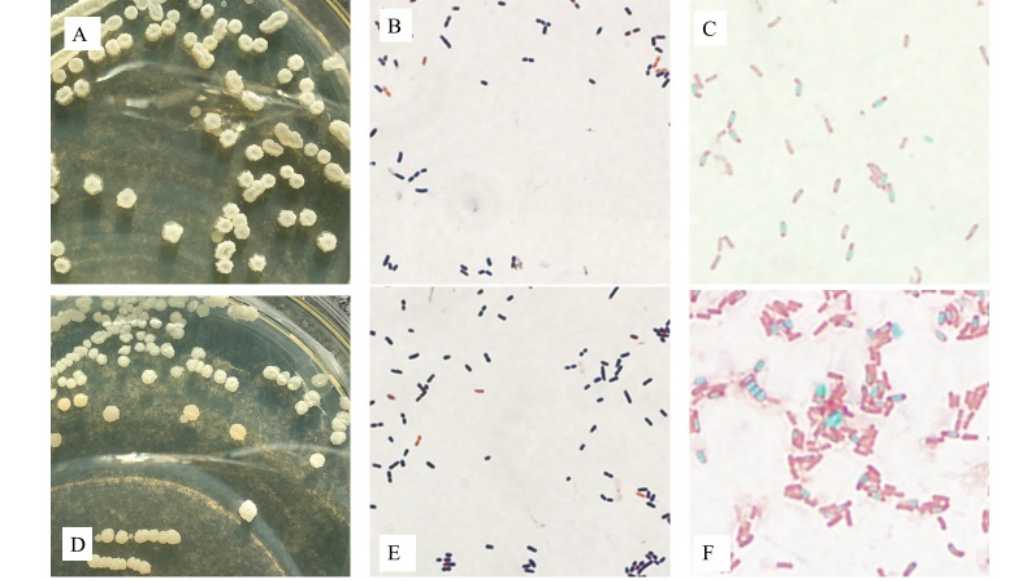

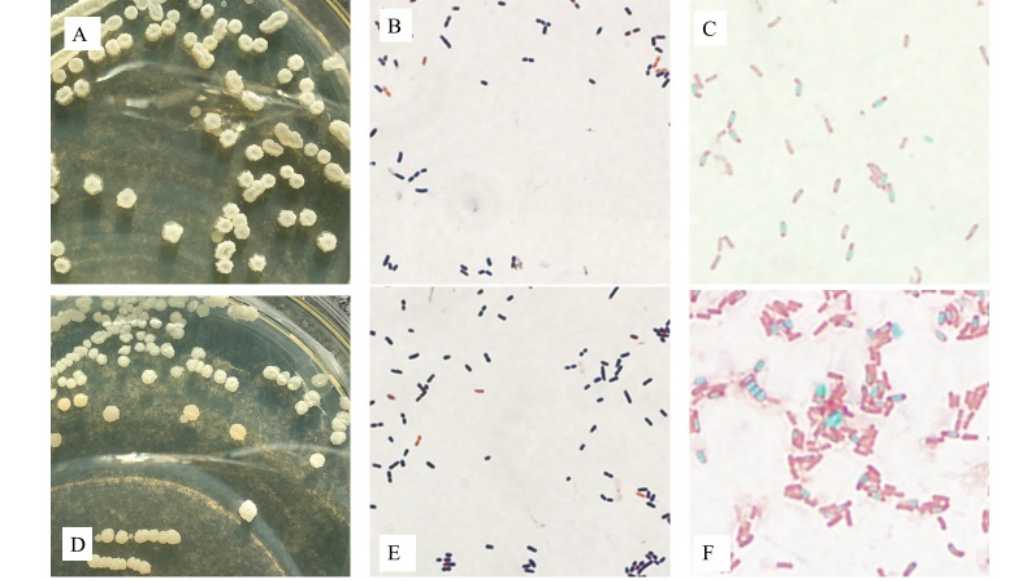


**d**

**c**

1000 ×

5 mm

**Fig.S2.** The morphological features of *B. velezensis* isolated from crucian carp intestine. **(A)** The colonies of *B. velezensis* isolates streaked on LB agar plates; **(B)** Microscopic analysis of Gram stain of *B. velezensis* strains, magnification: ×1,000; **(C)** Microscopic analysis of endospore stain of *B. velezensis* strains, magnification: ×1,000; (a) *B. velezensis* C-11; (b) *B. velezensis* S-22; (c) *B. velezensis* L-17; (d) *B. velezensis* S-14.


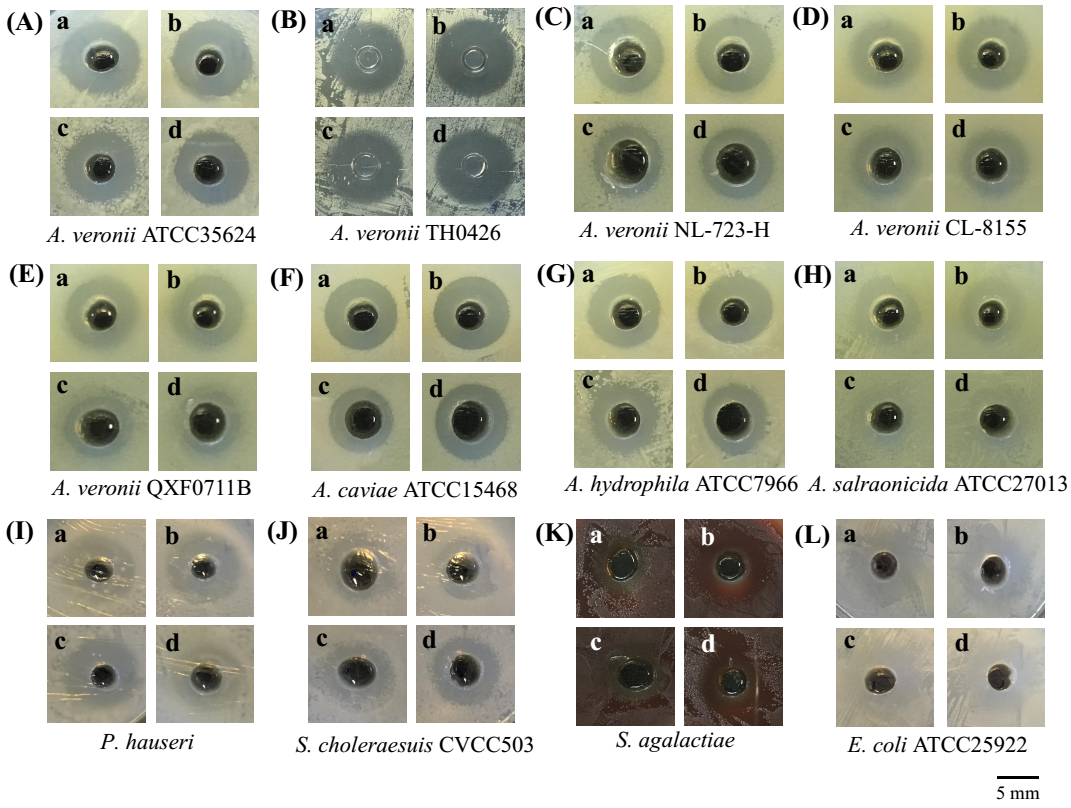
**Fig.S3.** Antimicrobial test of *B. velezensis* C-11 (a), S-22 (b), L-17 (c) and S-14 (d) against various pathogenic bacteria using paper disk infusion method. Strong antimicrobial activity was observed in *A. veronii* **(A, B, C, D&E)**, *A. caviae* **(F)**, *A. hydrophila* **(G)**, *A. salraonicida* **(H)**, *P. hauseri* **(I)**, *S. choleraesuis* **(J)**, *S. aureus* **(K)** *and E. coli* **(L)**.

**
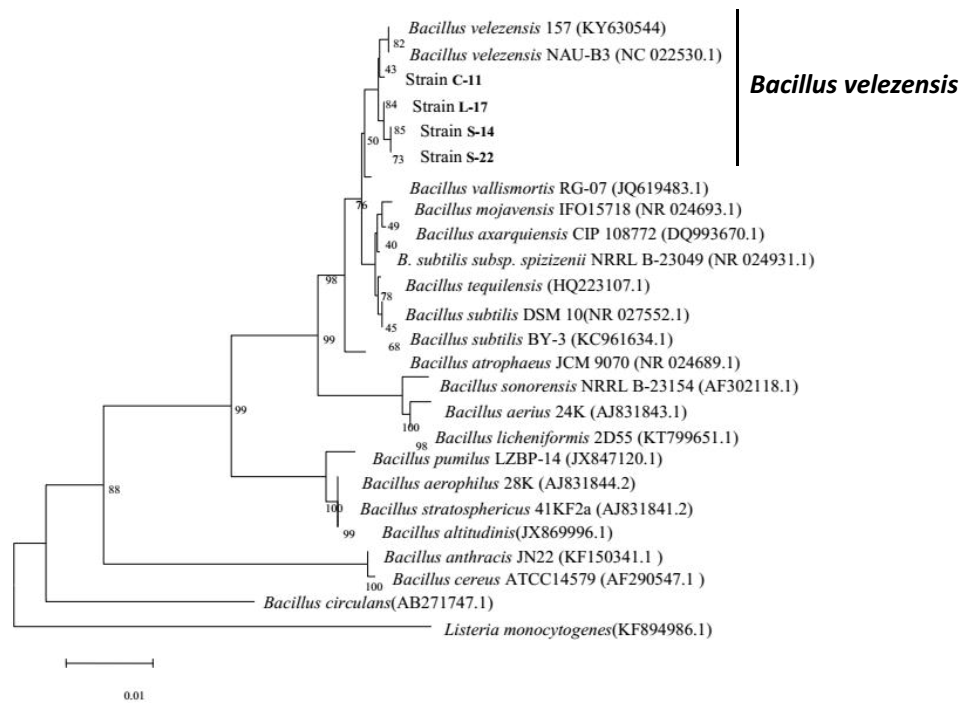
**

**Fig.S4.** Phylogenetic tree of the closely related species in *Bacillus* genus based on 16S rRNA gene sequences. The sequences were aligned using Clustal W tool in MEGA 7.0, and phylogenetic analysis was performed using the neighbor-joining method. The bootstrap percentages, based on 10,000 replications, are shown. The scale bars indicate 0.01 substitution per nucleotide position. The gene accession numbers are shown in the parentheses after each species.


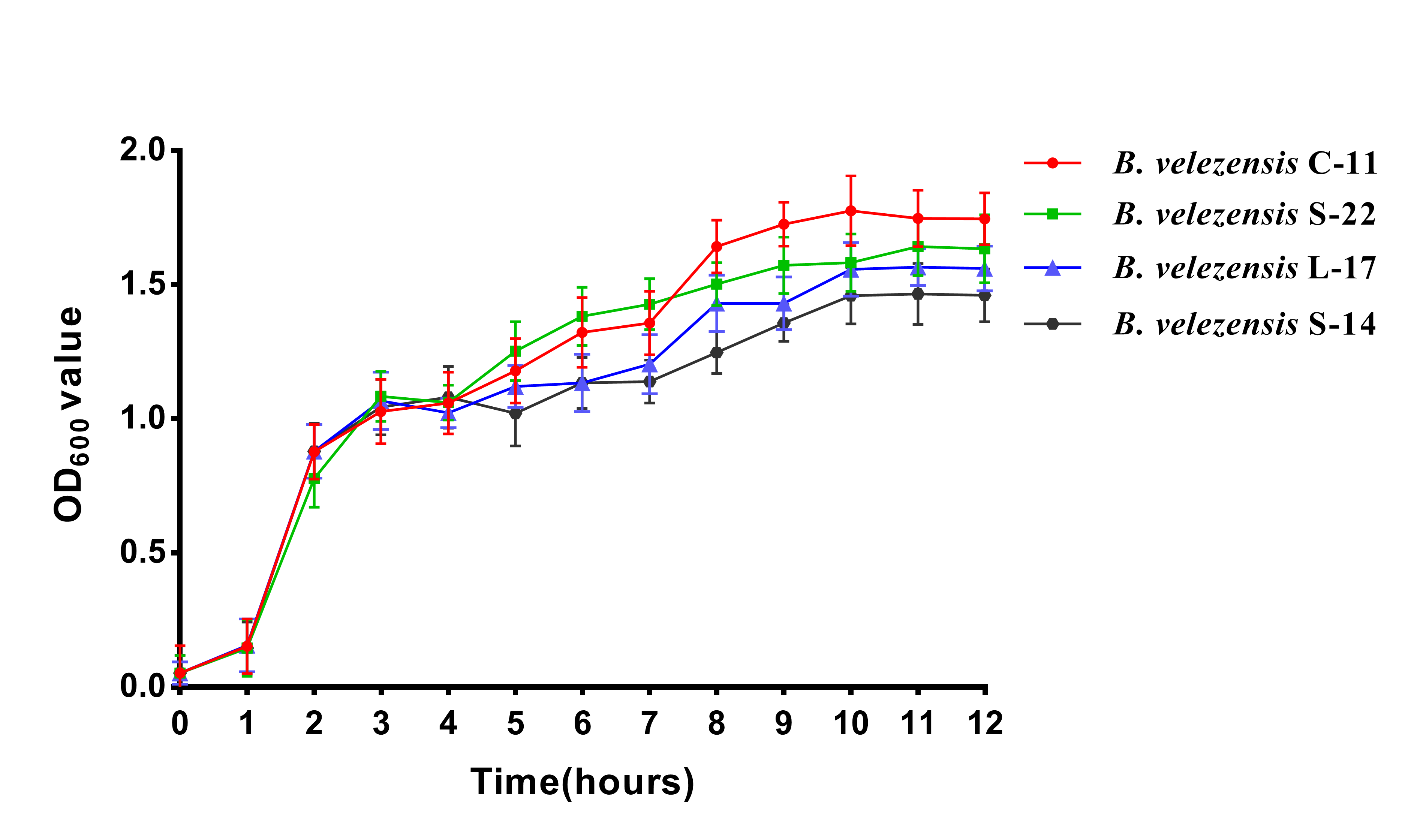


**Fig.S5.** Growth curves of the *B. velezensis* C-11 (red curve), S-22 (green curve), L-17 (blue curve) and S-14 (black curve). Each curve represents the results of 3 replicates.


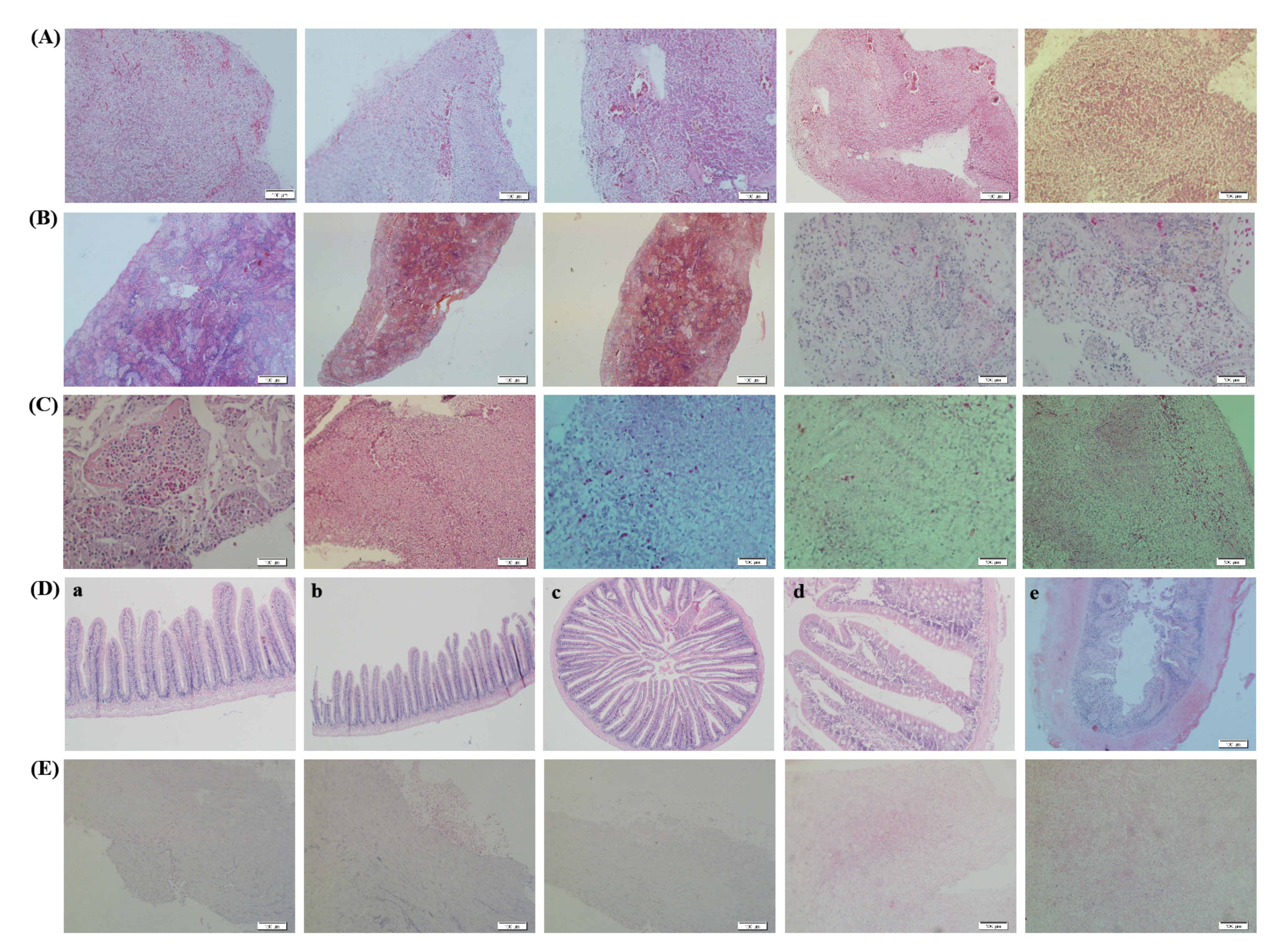


**Fig.S6.** Haematoxylin-eosin staining of spleen **(A)**, head kidney **(B)**, liver **(C)**, intestine **(D)** and heart **(E)** after administration of *B. velezensis*. *B. velezensis* C-11 (a), S-22 (b), L-17 (c), S-14 (d) and control group (e). (Scale bars, 100 μm).

**Table S1.** Fermentation of carbohydrates by *Bacillis velezensis* strains tested by API50CHB test kits.

| Carbohydrates | Strain | | | | Carbohydrates | Strain | | | |
| --- | --- | --- | --- | --- | --- | --- | --- | --- | --- |
|  | C-11 | S-14 | L-17 | S-22 |  | C-11 S-14 L-17 S-22 | | | |
| Propanediol | + | + | + | + | Salicin | - | - | - | - |
| Dulcitpl | + | + | + | + | D-Cellobiose | + | - | - | - |
| L-Arabinose | - | - | - | - | D-Maltose | + | - | + | - |
| D-Arabinose | - | - | - | - | D-Lactose | - | - | - | - |
| D-Ribose | - | - | - | - | D-Melibiose | + | - | - | - |
| D-Xylose | + | + | + | + | D-Saccharose | - | - | - | - |
| L-Xylose | + | + | + | + | D-Trehalose | - | + | - | + |
| L-rhamnose monohydrate | - | - | - | - | D-Melezitose | - | - | - | - |
| D-Galactose | + | + | + | + | D-Raffinose | - | - | - | - |
| D-Fructose | + | + | + | + | Amidon | + | + | + | + |
| D-Mannose | + | - | - | + | Glycogen | + | + | + | + |
| Tryptophan | + | + | + | - | Xylitol | - | - | - | - |
| L-Rhamnose | - | - | - | - | Gentiobiose | - | - | - | - |
| Dulcitol | - | - | - | - | D-Turanose | - | - | - | - |
| Inositol | + | - | - | - | D-Lyxose | - | - | - | - |
| D-Mannitol | + | + | + | + | D-Tagatose | - | - | - | - |
| D-Sorbitol | - | - | + | - | D-Fucose | - | - | - | - |
| Methyla-D-mannopyranoside | - | - | - | - | L-Fucose | - | - | - | - |
| Methyl a-D-glucopyranoside | - | - | - | - | D-Arabitol | - | - | - | - |
| Potassium | - | - | - | - | Arbutin | - | - | + | + |
| Amygdalin | - | - | - | - |  |  |  |  |  |

+: Positive reaction; –: Negative reaction.

**Table S2.** Antibacterial activity of the isolated probiotic against fish pathogens and food borne pathogens.

| Strain | Diameter of inhibition zone (mm) | | | |
| --- | --- | --- | --- | --- |
|  | C-11 | S-14 | L-17 | S-22 |
| *Aeromonas veronii* ATCC35624 | 23 ± 0.1 | 21 ± 0.3 | 18 ± 0.3 | 15 ± 0.3 |
| *Aeromonas veronii* TH0426 | 24 ± 0.2 | 20 ± 0.1 | 21 ± 0.2 | 22 ± 0.1 |
| *Aeromonas veronii* 75 | 17 ± 0.3 | 13 ± 0.3 | 23 ± 0.1 | 19 ± 0.1 |
| *Aeromonas veronii* 115 | 18 ± 0.2 | 12 ± 0.3 | 19 ± 0.3 | 17 ± 0.2 |
| *Aeromonas veronii* NL-723-H | 19 ± 0.1 | 14 ± 0.2 | 16 ± 0.1 | 13 ± 0.3 |
| *Aeromonas veronii* CL-8155 | 15 ± 0.4 | 11 ± 0.3 | 18 ± 0.2 | 12 ± 0.1 |
| *Aeromonas veronii* QXF0711B | 17 ± 0.1 | 13 ± 0.1 | 18 ± 0.2 | 14 ± 0.3 |
| *Aeromonas veronii* JL-8155 | 19 ± 0.2 | 10 ± 0.3 | 17 ± 0.1 | 11 ± 0.2 |
| *Aeromonas hydrophila* ATCC7966 | 18 ± 0.1 | 12 ± 0.1 | 16 ± 0.2 | 14 ± 0.3 |
| *Aeromonas caviae* ATCC15468 | 16 ± 0.3 | 17 ± 0.4 | 18 ± 0.2 | 13 ± 0.1 |
| *Aeromonas salraonicida* ATCC27013 | 19 ± 0.2 | 14 ± 0.3 | 16 ± 0.2 | 12 ± 0.1 |
| *Streptococcus agalactiae* | 27 ± 0.2 | 17 ± 0.1 | 20 ± 0.3 | 14 ± 0.2 |
| *Staphylococcus aureus* CVCC519 | 10 ± 0.4 | 11 ± 0.3 | 11 ± 0.1 | 12 ± 0.1 |
| *Escherichia coli* ATCC25922 | 18 ± 0.1 | 16 ± 0.1 | 15 ± 0.3 | 14 ± 0.2 |
| *Escherichia coli* BNCC125988 | 11 ± 0.1 | 12 ± 0.3 | 10 ± 0.1 | 13 ± 0.2 |
| *Escherichia coli* CVCC233 | 10 ± 0.3 | 19 ± 0.4 | 11 ± 0.2 | 13 ± 0.1 |
| *Escherichia coli* ATCC236 | 22 ± 0.2 | 15 ± 0.1 | 18 ± 0.1 | 16 ± 0.3 |
| *Salmonella Typhimurium* ATCC25241 | 19 ± 0.1 | 16 ± 0.2 | 14 ± 0.2 | 17 ± 0.3 |
| *Salmonella choleraesuis* CVCC503 | 16 ± 0.3 | 17 ± 0.4 | 14 ± 0.1 | 12 ± 0.1 |
| *Salmonella choleraesuis* CVCC3378 | 18 ± 0.3 | 19 ± 0.3 | 16 ± 0.2 | 12 ± 0.2 |
| *Clostridium perfringens* | 20 ± 0.1 | 21 ± 0.3 | 17 ± 0.2 | 16 ± 0.3 |
| *Proteus hauseri* | 23 ± 0.2 | 22 ± 0.4 | 18 ± 0.3 | 14 ± 0.1 |

Inhibition zone expressed as external diameter minus inner diameter, values represent mean ± standard deviation (SD).

**Table S3.** Antibiotic susceptibility test of the *Bacillus* isolates C-11, S-14, L-17 and S-22.

| Antibiotics (μg) | Probiotic isolates | | | |
| --- | --- | --- | --- | --- |
|  | C-11 | S-14 | L-17 | S-22 |
| Ampicillin (10) | ++ | +++ | +++ | +++ |
| Piperacillin (10) | +++ | ++ | +++ | +++ |
| Cephalexin (30) | +++ | +++ | ++ | +++ |
| Cefazolin (15) | +++ | +++ | +++ | +++ |
| Cefradine (30) | +++ | +++ | +++ | +++ |
| Cefuroxim (30) | +++ | +++ | +++ | +++ |
| Ceftazidime (30) | +++ | ++ | +++ | +++ |
| Amikacin (10) | +++ | ++ | +++ | +++ |
| Gentamicin (10) | +++ | +++ | +++ | ++ |
| Kanamycin (10) | +++ | +++ | +++ | +++ |
| Neomycin (10) | +++ | +++ | +++ | +++ |
| Tetracycline (30) | ++ | +++ | ++ | ++ |
| Doxycycline (10) | +++ | +++ | +++ | +++ |
| Minocycline (10) | +++ | +++ | +++ | +++ |
| Erythromycin (15) | +++ | +++ | +++ | +++ |
| Norfloxacin (10) | +++ | ++ | +++ | +++ |
| Ofloxacin (10) | +++ | +++ | ++ | +++ |
| Ciprofloxacin (10) | +++ | +++ | +++ | +++ |
| Polymyxin B (30) | R | +++ | ++ | R |
| Furazolidone (10) | +++ | +++ | +++ | +++ |
| Chloramphenicol (30) | +++ | +++ | +++ | +++ |
| Amoxicillin (10) | +++ | +++ | +++ | +++ |
| Clindamycin (15) | +++ | +++ | +++ | +++ |

++ zone of inhibition between 15 and 19 mm, +++ zone of inhibition above 20 mm, R resistant.

**Table S4.** Effects of *A. veronii* on the intestinal villi of length, width, number per villus of goblet and inflammatory cells after orally administration with *Bacillus velezensis* strains.

| Groups | Intestinal villus | | Number per villus | Number per mm^2^ |
| --- | --- | --- | --- | --- |
|  | Length (μm) | Width (μm) | Goblet cells | Inflammatory cells |
| Blank Control | 266.93 ± 8.79^a^ | 56.00 ± 10.47^a^ | 18.5 ± 5.22^a^ | 42.43 ± 9.14^b^ |
| *A.veronii* | 179.94 ± 15.94^c^ | 72.97 ± 11.65^b^ | 12.5 ± 6.43^a^ | 80.57 ± 13.31^a^ |
| *B.velezensis* C-11+ *A.veronii* | 315.55 ± 42.45^a^ | 61.15 ± 6.28^a^ | 17.3 ± 11.10^b^ | 35.43 ± 11.84^b^ |
| *B.velezensis* S-22+ *A.veronii* | 232.88 ± 16.88^bc^ | 63.08 ± 11.87^a^ | 18.8 ± 4 .10^a^ | 37.71 ± 8.50^b^ |
| *B.velezensis* L-17+ *A.veronii* | 263.39 ± 35.41^ab^ | 67.64 ± 5.38^a^ | 24.7 ± 4.72^a^ | 51.00 ± 15.79^b^ |
| *B.velezensis* S-14+ *A.veronii* | 300.00 ± 49.10^a^ | 65.11±11.38^a^ | 22.8 ± 3.4^a^ | 62.43 ± 21.61^a^ |

Values are presented as mean ± SD (n = 6), and diﬀerent superscripts in the same column are significantly different (*P* < 0.05), the below is the same.
